# Firing Activities of REM- and NREM-Preferring Neurons Are Differently Modulated by Fast Network Oscillations and Behavior in the Hippocampus, Prelimbic Cortex, and Amygdala

**DOI:** 10.1523/ENEURO.0575-24.2025

**Published:** 2025-05-23

**Authors:** Risa Kajiya, Hiroyuki Miyawaki, Hirokazu Nakahara, Kenji Mizuseki

**Affiliations:** ^1^Department of Physiology, Osaka Metropolitan University Graduate School of Medicine, Osaka 545-8585, Japan; ^2^Department of Oral and Maxillofacial Surgery, Osaka Metropolitan University Graduate School of Medicine, Osaka 545-8585, Japan; ^3^Department of Physiology, Osaka City University Graduate School of Medicine, Osaka 545-8585, Japan; ^4^Department of Oral and Maxillofacial Surgery, Osaka City University Graduate School of Medicine, Osaka 545-8585, Japan

**Keywords:** cortical ripples, inter-regional coactivation, non-REM sleep, REM sleep, sharp-wave ripples, spindles

## Abstract

Sleep consists of two alternating states—rapid eye movement (REM) and non-REM (NREM) sleep. Neurons adjust their firing activity based on brain state, however, the extent to which this modulation varies across neurons and brain regions remains poorly understood. This study analyzed previously acquired 17-h continuous recordings of single-unit activity and local field potentials in the ventral hippocampal CA1 region, prelimbic cortex layer 5, and basolateral nucleus of the amygdala of fear-conditioned rats. The findings indicate that more than half of the neurons fired faster during REM sleep than during NREM sleep, although a notable subset of neurons exhibited the opposite preference, firing preferentially during NREM sleep. During sleep, the overall firing activity of both REM- and NREM-preferring neurons decreased. However, fast network oscillations, including hippocampal sharp-wave ripples (SWRs), amygdalar high-frequency oscillations, cortical ripples, and cortical spindles, differentially modulated REM- versus NREM-preferring neurons. During wakefulness, REM-preferring neurons fired more slowly but were more intensely activated by SWRs and shock presentations than NREM-preferring neurons. Moreover, during fast network oscillations in sleep, neurons with similar REM/NREM preferences exhibited stronger within- and cross-regional coactivation than those with differing preferences. Conversely, during awake SWRs in fear conditioning sessions, neurons with different REM/NREM preferences showed stronger interregional coactivation than those with similar preferences. These findings suggest that the distinct activity patterns of REM- and NREM-preferring neurons, potentially reflecting different roles in memory, affect local and global networks differently, thereby balancing experience-dependent network modifications with sleep-dependent homeostatic regulation of neuronal excitability.

## Significance Statement

Sleep consists of rapid eye movement (REM) and non-REM (NREM) states, each characterized by distinct neuromodulatory tones, network dynamics, and memory-related functions. However, how REM and NREM sleep regulate neuronal firing remains unclear. This study reveals that neurons exhibit diverse sleep-state firing preferences, with both REM- and NREM-preferring neurons similarly downregulated during sleep but differentially modulated by fast network oscillations. Neurons with similar REM/NREM preferences showed stronger intra- and inter-regional coactivation during sleep, while those with differing preferences exhibited stronger inter-regional coactivation during emotionally salient experiences. These findings suggest that REM- and NREM-preferring neurons play distinct roles in shaping local and global networks, potentially balancing experience-dependent network modifications with sleep-dependent homeostatic regulation of neuronal excitability.

## Introduction

Sleep is broadly categorized into two alternating states—rapid eye movement (REM) and non-REM (NREM) sleep—each thought to play distinct roles in memory processing (Buzsáki, 1989; Diekelmann and Born, 2010; Rasch and Born, 2013; Boyce et al., 2016; Izawa et al., 2019; Kumar et al., 2020). These states are characterized by distinct neuromodulatory environments and neural network dynamics, reflecting different patterns of synaptic inputs (Hobson and Pace-Schott, 2002; Niethard et al., 2016). Neurons exhibit variable responses to neuromodulators and synaptic inputs (Koulakov et al., 2009; Schmidt et al., 2013), suggesting that sleep-state modulation of neuronal firing differs across cell populations. On average, neocortical neurons tend to be more active during REM than during NREM sleep (Vyazovskiy et al., 2009; Watson et al., 2016); additionally, hippocampal neurons fire at higher rates during REM than during NREM sleep (Grosmark et al., 2012; Miyawaki and Diba, 2016; Miyawaki et al., 2019). However, the extent to which firing rates (FRs) are differentially modulated by REM and NREM sleep across individual neurons remains unclear.

Sleep is essential for restoring brain function. The synaptic homeostasis hypothesis suggests that sleep reduces synaptic strengths that are augmented during wakefulness (Tononi and Cirelli, 2003). Supporting this hypothesis, average neuronal activity in the cortex and hippocampus declines across sleep (Vyazovskiy et al., 2009; Grosmark et al., 2012; Miyawaki and Diba, 2016; Watson et al., 2016). Sharp-wave ripples (SWRs), hippocampal fast network oscillations (100–250 Hz), contribute to the induction of long-term depression (LTD) of synapses (Norimoto et al., 2018) and the downscaling of neuronal activity (Miyawaki and Diba, 2016). Memory consolidation is another well-established function of sleep (Diekelmann and Born, 2010), wherein SWRs play a crucial role (Wilson and McNaughton, 1994; Lee and Wilson, 2002; Girardeau et al., 2009; Ego-Stengel and Wilson, 2010; Girardeau and Zugaro, 2011), presumably by inducing synaptic long-term potentiation (LTP; Buzsáki et al., 1987; King et al., 1999). Thus, SWRs can induce both LTD and LTP during sleep. The direction of synaptic weight changes depends on the relative timing of pre- and postsynaptic neuronal firing (Markram et al., 1997; Bi and Poo, 1998). During NREM sleep, the high-FR cortical neurons tend to fire earlier than the low-FR cortical neurons during transition from brief (30–200 ms) population-wide silent state (DOWN state) to sustained activity state (UP state; Peyrache et al., 2010; Watson et al., 2016). This FR-dependent sequence may strengthen synapses from earlier firing (high-FR) to later firing (low-FR) neurons while weakening synapses in the opposite direction (Wei et al., 2016), thereby narrowing the FR distribution (Levenstein et al., 2017). A similar pattern is observed during SWRs, where high-FR pyramidal neurons and interneurons in the hippocampal CA1 region fire earlier than their low-FR counterparts (Fernández-Ruiz et al., 2019). Although most hippocampal neurons exhibit increased firing during SWRs, the extent of this enhancement varies across neurons (Mizuseki and Buzsáki, 2013; Mizuseki and Miyawaki, 2023). This indicates that FR modulation by SWRs for a given neuron may influence the direction of synaptic weight changes during sleep.

Amygdalar high-frequency oscillations (HFOs, 90–180 Hz; Ponomarenko et al., 2003; Cox et al., 2020; Perumal et al., 2021), cortical ripples (cRipples, 90–180 Hz; Grenier et al., 2001; Khodagholy et al., 2017), and cortical spindles (9–18 Hz; Steriade et al., 1993; De Gennaro and Ferrara, 2003) are prominent during NREM sleep, although they also occur during REM sleep and wakefulness (Khodagholy et al., 2017; Bandarabadi et al., 2020). Hippocampal SWRs are temporally coupled with these oscillations (Maingret et al., 2016; Khodagholy et al., 2017; Miyawaki and Mizuseki, 2022), facilitating coordinated activity across brain regions essential for memory consolidation and integration (Maingret et al., 2016; Khodagholy et al., 2017; Latchoumane et al., 2017; Klinzing et al., 2019; Cox et al., 2020; Girardeau and Lopes-dos-santos, 2021). In addition, spindles, like SWRs, are associated with the downscaling of neural activity during sleep (Miyawaki and Diba, 2016). However, whether FR modulation by sleep states correlates with that by fast network oscillations remains unclear.

A previous study (Miyawaki and Mizuseki, 2022) employed simultaneous electrophysiological recordings from the ventral hippocampal CA1 region (vCA1), prelimbic cortex layer 5 (PL5), and basolateral nucleus of the amygdala (BLA) in fear-conditioned rats, demonstrating enhanced cross-regional coactivation of local neuronal populations following fear conditioning. However, the extent to which individual neuronal activity is modulated by sleep state—and how this modulation relates to within- and cross-regional coactivations—remains unclear. To address these questions, the present study analyzed previously collected data (Miyawaki and Mizuseki, 2022) to investigate variability in REM/NREM preference and firing modulation by fast network oscillations across neurons in the vCA1, PL5, and BLA. The findings revealed significant variation in REM/NREM preference, which correlated with firing modulation during fast network oscillations in sleep, awake SWRs, and emotionally salient experiences. Moreover, during fast network oscillations in sleep, neurons with similar REM/NREM preferences exhibited stronger within- and cross-regional coactivation than those with different preferences. In contrast, during awake SWRs in fear conditioning sessions, neurons with divergent REM/NREM preferences showed stronger within-regional coactivation than those with similar preferences.

## Materials and Methods

In a previous study (Miyawaki and Mizuseki, 2022), single-unit activity and local field potentials (LFPs) were recorded from vCA1, PL5, and BLA, along with electromyography (EMG), electrocardiography (ECG), and electro-olfactography (EOG), in male Long–Evans rats (*n* = 15). However, that study did not systematically examine how individual neuronal firing is modulated by sleep states and fast network oscillations. Cell type classification, sleep scoring, and detection of fast network oscillations were performed using previously published MATLAB scripts (Miyawaki and Mizuseki, 2022; https://github.com/HiroMiyawaki/Miyawaki2022_NatCommun, https://doi.org/10.5281/zenodo.6069800). The analytical methods and relevant experimental procedures are described below, including those outlined in the original publication.

### Animals, surgery, and data collection

All animal procedures followed National Institutes of Health guidelines and were approved by the Institutional Animal Care and Use Committee of Osaka City University (approval #15030). Fifteen male Long–Evans rats (9.6–15.0 weeks old, 330–503 g at the time of surgery, Japan SLC) were used. Animals were housed on a 12 h light/dark cycle (lights on at 8:00 A.M.) with *ad libitum* access to food and water.

For surgical procedures, rats were anesthetized with isoflurane (1–3% in a 50% air/50% oxygen mixture). Stainless steel wires for EMG and ECG recording, screws for EOG recording, reference and ground electrodes, and tungsten wires for eyelid stimulation were implanted. Three craniotomies were performed, centered at the following coordinates relative to bregma: anterior–posterior (AP) +2.90 to +3.25 mm, medial–lateral (ML) +1.0 to +1.5 mm; AP −2.60 to −3.00 mm, ML +4.60 to +4.80 mm; and AP −4.95 to −5.55 mm, ML +2.80 to +3.00 mm. Silicon probes (Buzsaki64sp and Buzsaki64spL from NeuroNexus or F6-64 from Cambridge Neurotech) were implanted through the cranial windows targeting the right PL5, BLA, and vCA1. Electrode positions were confirmed by histological reconstruction and previously reported in detail (Miyawaki and Mizuseki, 2022).

Following 3–15 d of recovery, animals were connected to a recording system (C3100 256-ch acquisition board from Intan or 512-ch acquisition board from Open Ephys). Signals from all probes, along with EMG, ECG, and EOG, were continuously recorded for ∼17 h (start: 21:31–21:47 [median = 21:37]; end: 14:24–14:27 [median = 14:26], Zeitgeber time) during the course of fear conditioning.

**Figure 1.**
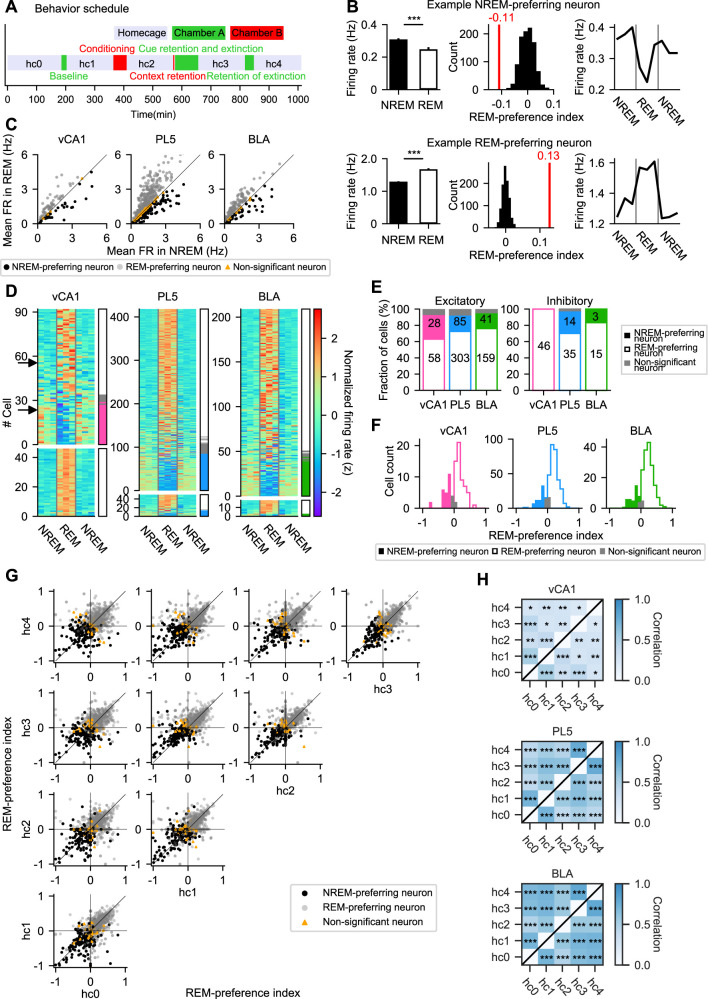
NREM- and REM-preferring neurons in the vCA1, PL5, and BLA. ***A***, Schematic of the experimental schedule. Adapted from Miyawaki and Mizuseki (2022) under a CC-BY 4.0 license. ***B***, Mean firing rates (FRs) during NREM and REM sleep (left), REM-preference indices (red line in middle plots) with distributions of shuffled surrogates (histograms, bin label shuffling, *n* = 1,000), and mean FRs across NREM–REM–NREM triplets (right, *n* = 23 triplets for both example neurons) for two representative excitatory neurons in the vCA1. Neurons with REM-preference indices below the 2.5th percentile or above the 97.5th percentile of the surrogate distribution were classified as NREM-preferring (top) or REM-preferring (bottom), respectively. ***p < 0.001; *p* = 4.1 × 10^−13^ (top), *p* = 2.6 × 10^−33^ (bottom); Mann–Whitney *U* test. ***C***, Comparison of mean FRs during NREM and REM sleep among NREM-preferring (black circles), REM-preferring (gray circles), and non-significant (orange triangles) excitatory neurons in the vCA1, PL5, and BLA. ***D***, Mean FRs of all analyzed neurons in the vCA1, PL5, and BLA during NREM–REM–NREM triplets. FRs were *Z-*scored within each neuron; neurons are sorted by REM-preference indices. Excitatory and inhibitory neurons are shown above and below the white lines, respectively. Black arrows indicate the neurons shown in ***B***. Bars on the right indicate NREM-preferring (colored), REM-preferring (hollow), and non-significant (gray) neurons. ***E***, Proportions of NREM-preferring (colored), REM-preferring (hollow), and non-significant (gray) neurons among excitatory (left) and inhibitory (right) neurons. The numbers of NREM- and REM-preferring neurons are superimposed on each bar. ***F***, Distributions of REM-preference indices for excitatory neurons in the vCA1, PL5, and BLA. REM-preference indices were calculated using all sleep epochs across all home cage (hc) and behavioral sessions. ***G***, Comparisons of REM-preference indices for excitatory neurons calculated within each hc session. REM/NREM preferences were largely consistent across sessions. Each dot represents a single neuron. All excitatory neurons in the vCA1, PL5, and BLA are plotted together. Dot colors indicate neuron classification based on all concatenated sleep epochs, as in ***C–F***. NREM-preferring (black), REM-preferring (gray), and non-significant (orange). For separate plots for each region, see Extended Data Figure 1-4. ***H***, Spearman's rank-order correlation coefficients (*ρ*) of REM-preference indices across hc sessions for excitatory neurons in each region. All excitatory neurons in the vCA1, PL5, and BLA, including non-significant neurons, were included in the correlation analyses. *p < 0.05, **p < 0.01, ***p < 0.001. The number of neurons analyzed in ***C–H*** is summarized in Table 1. The total numbers of NREM–REM–NREM triplets used in ***D*** are reported in Extended Data Figure 1-2. Statistical details of ***H*** are summarized in Extended Data Figure 1-5.

The fear conditioning experiment consisted of five behavioral sessions—baseline, conditioning, context-retention, cue-retention/extinction, and retention of extinction. The baseline, cue-retention/extinction, and retention of extinction sessions were conducted in a rectangular box (27 × 33 cm; depth, 40 cm), while the conditioning and context-retention sessions were conducted in a cylindrical tube (30 cm diameter, 40 cm depth). Briefly, 30-s, 5 kHz pips (250 ms on, 750 ms off, 74 dB) were used as conditioned stimuli (CS), and 2-s electric shocks (2-ms pulses at 8 Hz, alternating between left and right eyelids) were used as unconditioned stimuli (US). In the context-retention session, rats were returned to their home-cage immediately after the 4-min free-exploration period. In other behavioral sessions, the rats received the first CS after 4 min of context exposure, receiving 4, 12, 40, and 8 CSs in the baseline, conditioning, cue-retention/extinction, and retention of extinction sessions, respectively. CSs were delivered at random intervals of 180–240 s, except for the last 32 CSs in the cue-retention/extinction session, which were delivered at uniformly distributed intervals of 60–120 s. In the conditioning session, USs were delivered 700–750 ms after each CS offset. Between behavioral sessions, rats were returned to their home cages (hc sessions). These sessions were designated as hc0 (before the baseline session), hc1 (between baseline and conditioning), hc2 (between conditioning and context-retention), hc3 (between cue-retention/extinction and retention of extinction), and hc4 (after retention of extinction; Fig. 1*A*). Unless otherwise noted, data from all hc sessions were pooled for analysis.

### Spike sorting and cell type classifications

Eyelid shock artifacts were estimated using a third-order Savitzky–Golay filter equipped in MATLAB and subtracted from the recorded traces from 10 ms before shock onset to 1 s after shock offset. Subsequently, automatic spike sorting was performed using Kilosort2 (Stringer et al., 2019; Pachitariu et al., 2024; http://github.com/MouseLand/Kilosort2), followed by manual curation with Phy (http://github.com/cortex-lab/phy). To prevent artifact contamination, spikes occurring from 0.1 ms before the onset to 5 ms after the offset of each 2-ms shock pulse were discarded. Cluster quality was assessed using the following metrics (Miyawaki and Mizuseki, 2022): Isolation distance (Harris et al., 2001) was calculated with clusters detected on the same shank. Inter-spike interval (ISI) index (Fee et al., 1996) was calculated using the formula $\frac{\mathrm{IS}I_{0.5-2}}{\mathrm{IS}I_{2-10}}\times \frac{8}{1.5}$, where ISI_0.5–2_ and ISI_2–10_ are the counts of ISI in [0.5, 2] ms and [2, 10] ms windows, respectively. Since the ISI index is unsuitable for certain nonbursty cells, the contamination rate calculated in Kilosort2 was also used, as follows:

$$\min\left(\frac{\mathrm{AC}G_{0.5-n}}{\mathrm{AC}G_{0.5-49.5}}\times \frac{49}{n\;-0.5},\;\frac{\mathrm{AC}G_{0.5-n}}{\mathrm{AC}G_{250-500}}\times \frac{250}{n\;-0.5}\right),$$

where ACG*_x-y_* is the count of the spike auto-correlogram in an [*x*, *y*] ms window, and the minimum value was found by shifting *n* from 1.5 to 9.5 ms in 1 ms steps.

The mean waveform for each cluster was extracted from high-pass filtered traces (>300 Hz). The channel with the largest spike amplitude was identified, and its mean waveform was upsampled to 200 kHz using the spline function in MATLAB (MathWorks). Spike amplitude (trough depth from baseline) and spike width (trough-to-peak duration) were measured from this upsampled mean waveform. Clusters were included in further analysis only if they met all the following four criteria: (1) isolation distance >15, (2) ISI index <0.2 or contamination rate <0.05, (3) overall mean FRs >0.01 Hz, and (4) spike amplitude >50 μV.

Putative excitatory and inhibitory neurons were identified by analyzing monosynaptic connectivity, following previously described methods (Fujisawa et al., 2008) with minor modifications (Miyawaki and Mizuseki, 2022). For neuron pairs recorded within the same brain region (vCA1, PL5, or BLA), spike timing cross-correlograms (CCGs) were computed using 0.1 ms bins and smoothed with a Gaussian kernel (*σ* = 0.5 ms). To generate a surrogate distribution, random jitters distributed uniformly in the range of [−5, +5] ms were added for each spike for each cell independently, then the CCG was calculated using the same procedure as the original spike trains. The maximum and minimum of CCG values in the range of [−5, +5] ms were detected. This process was repeated 1,000 times to construct the 99% global bands (i.e., the 0.5th and 99.5th percentiles of these maxima and minima). If the actual smoothed CCG had a significant peak or trough with respect to the 99% global bands in [+1, +4] ms periods, the cell pair was considered as a candidate exhibiting a monosynaptic excitatory or inhibitory connection, respectively (Extended Data Table 1-1*A*). Pairs with suspicious connections (e.g., broad or zero-lag peaks/troughs) were manually excluded, and the remaining pairs were classified as monosynaptically connected. Cells forming only excitatory or inhibitory outputs (but not both) were labeled as excitatory or inhibitory neurons, respectively. Nearly all excitatory neurons classified by CCG had spike width >0.6 ms, while inhibitory neurons had width <0.5 ms (Miyawaki and Mizuseki, 2022). Thus, following Barthó et al. (2004), cells that could not be classified by CCGs were classified based on spike width (>0.6 ms = excitatory, <0.5 ms = inhibitory), and any remaining cells were excluded from analysis (Extended Data Table 1-1*B*; Miyawaki and Mizuseki, 2022).

### Sleep scoring

Sleep states were automatically detected in 1-s windows using Buzcode scripts (http://github.com/buzsakilab/buzcode), with minor manual adjustments based on visual inspection of power spectra of LFPs of the prelimbic cortex and ventral hippocampus as well as EMG signals. Microarousal, brief (<40 s) arousal periods during NREM sleep or during the transition from REM to NREM sleep (Watson et al., 2016; Miyawaki et al., 2019), were treated as part of NREM sleep (Miyawaki and Mizuseki, 2022). NREM, REM, and wakefulness epochs lasting ≤50 s were excluded from the analyses. “Extended sleep” was defined as uninterrupted sleep lasting >30 min, without any wake epoch longer than 60 s (Miyawaki and Diba, 2016; Torrado Pacheco et al., 2021). The NREM–REM–NREM triplet was defined as three consecutive NREM, REM, and NREM epochs, each lasting >50 s (Grosmark et al., 2012; Miyawaki and Diba, 2016). The durations of NREM and REM sleep and wakefulness during the hc and behavioral sessions are summarized in Extended Data Figure 1-1. Unless otherwise specified, NREM, REM, and wakefulness epochs from all hc and behavioral sessions were pooled for subsequent analysis.

### Detection of SWRs, HFOs, cRipples, and cortical spindles

HFOs, cRipples, and cortical spindles were detected exclusively during NREM sleep, as these network oscillations are rarely observed during REM sleep (Steriade et al., 1993; Grenier et al., 2001; De Gennaro and Ferrara, 2003; Ponomarenko et al., 2003; Khodagholy et al., 2017; Cox et al., 2020). In contrast, SWRs were detected during both NREM sleep and wakefulness but not during REM sleep, as they typically occur in quiet wakefulness and NREM sleep (Buzsáki, 2015). SWRs were detected in the LFPs of the ventral hippocampus using a previously described method (Miyawaki and Mizuseki, 2022). Detection of ripples and sharp-waves was performed separately, and ripples co-occurring with sharp-waves were classified as SWRs (Liu et al., 2022). For each channel, ripples were detected using the root mean square (RMS) of bandpass filtered (100–250 Hz) LFPs calculated in 13.3 ms time windows. The RMS was *Z*-scored based on the mean and standard deviation (SD) during NREM epochs, and ripple candidates were identified as periods during which the RMS was >1.5 *z*. Ripple candidates with peak <4 *z* or duration <30 ms were discarded, and those separated by <10 ms were merged. Overlapping ripple candidates detected on the same shank were concatenated. Sharp-waves were detected based on the differences between the most superficial and deepest channels on the same shank. The signal were bandpass filtered (2–40 Hz), *Z*-scored using the mean and SD during NREM epochs, and sharp-wave candidates were defined as periods with signals <−2.5 *z*. Candidates lasting 20–400 ms were classified as sharp-waves. Ripple candidates during which at least one sharp-wave trough was detected on the same shank were accepted as candidate SWRs. In addition, overlapping candidate SWRs detected on different shanks were concatenated, and the resultant candidates with duration <750 ms were considered as SWRs.

HFOs and cRipples were detected using a previously described method (Miyawaki and Mizuseki, 2022). Briefly, HFOs were identified using the median LFPs for each shank in the amygdala, while cRipples were detected on individual channel LFPs in the prelimbic cortex. The power (RMS) of the bandpass filtered (90–180 Hz) LFPs was calculated in 20 ms time windows and then normalized to a *Z*-score based on the mean and SDs during NREM epochs. Periods with power >2 *z* were marked as HFO candidates. A candidate was classified as an HFO if its peak power was ≥4 *z* and duration was ≥30 ms. HFOs separated by <20 ms were merged, and overlapping events were concatenated across shanks. cRipples were identified as periods with power >3 *z*. Candidates were confirmed as cRipples if the peak power was ≥5 *z* and the duration was ≥50 ms. cRipples separated by <30 ms were merged, and overlapping events across different channels were concatenated. HFOs and cRipples longer than 750 ms were excluded from the analyses.

Spindles were detected using the wavelet power of the LFPs in the prelimbic cortex, following a previously established method (Sullivan et al., 2014). Because spindles are recorded coherently across multiple channels/shanks, the discrete wavelet transform was applied to the average LFP across all prelimbic cortical channels (in the 9–18 Hz band, 11 scales) using the MATLAB wavelet software package (provided by C. Torrence and G. Compo; https://github.com/chris-torrence/wavelets). Wavelet power was *Z-*scored within each scale using means and SDs during NREM sleep. The highest normalized power among the 11 scales at each time point was designated as spindle power. Epochs with spindle power >1.4 *z* for >350 ms within NREM epochs were marked as candidate spindles. A spindle was confirmed if its maximum power was ≥2 *z*.

Different laboratories, and occasionally even the same laboratory, employ varying criteria to identify network oscillations such as SWRs (Liu et al., 2022). To assess the robustness of the findings against different detection criteria, all analyses were repeated using more stringent peak power thresholds (*z* > 5, 5, 6, and 3 for SWRs, HFOs, cRipples, and spindles, respectively). The results remained qualitatively similar (data not shown), supporting the reliability of the findings.

### Classification of REM- and NREM-preferring neurons

To classify neurons based on their firing preference during REM and NREM sleep, FRs of each cell were first calculated in 1-s bins. The mean FRs across REM (FR*_R_*) and NREM (FR*_N_*) bins were then computed. Sleep-state preference was assessed using the REM-preference index, defined as follows:

$$\mathrm{REM}\text{-}\mathrm{preference}\;\mathrm{index}\;=\frac{FR_{R}-FR_{N}}{FR_{R}+FR_{N}}.$$

To determine whether the REM-preference index of a neuron significantly deviated from 0, a distribution of REM-preference indices was generated from shuffled surrogate data (REM/NREM bin label shuffling, *n* = 1,000) for each neuron (Fig. 1*B*, middle panels). Neurons with actual REM-preference indices >97.5th percentile or <2.5th percentile of the surrogate distribution were classified as REM- or NREM-preferring, respectively; all others were categorized as non-significant. To evaluate the robustness of this classification, more stringent thresholds were also applied (i.e., >99.5th or <0.5th percentile and >99.9th or <0.1st percentile). The classification results remained largely unchanged (Extended Data Fig. 1-3), supporting the reliability of the method.

To visualize the diversity of REM/NREM preferences across neurons (Fig. 1*B*,*D*), the FR of each neuron was computed within each third of NREM and REM epochs in NREM–REM–NREM triplets. The mean FR across all detected triplets was then calculated.

To assess whether the correlations of REM-preference indices significantly deviated from zero while controlling for differences in the number of recorded neurons, bootstrap analyses were conducted. For each combination of hc sessions within each brain region, an equal number of neurons (*n* = 92; corresponding to the minimum number of excitatory neurons recorded in vCA1, PL5, and BLA) were resampled with replacement. Spearman's rank-order correlation coefficients were then computed for REM-preference indices between the hc sessions. This resampling procedure was repeated 5,000 times to estimate the correlation coefficients and assess their statistical significance (Extended Data Figs. 1-6, 1-7).

The same bootstrap strategy was employed to determine whether correlation strengths varied across temporally adjacent hc session pairs. For each combination of temporally adjacent hc session pairs, differences in correlation coefficients were computed based on resampled data (*n* = 92). This procedure was repeated 5,000 times to estimate the 95% confidence intervals (CIs) and assess the statistical significance of the differences (Extended Data Figs. 1-8*A*, 1-9).

Similarly, to test for regional differences in REM-preference index correlations, the same bootstrap method was applied. For each pair of temporally adjacent hc sessions, correlation coefficients were computed separately for each brain region using resampled neurons (*n* = 92), and inter-regional differences were calculated. This process was repeated 5,000 times to generate 95% CIs and evaluate the statistical significance of the differences (Extended Data Figs. 1-8*B*, 1-9).

### FR gain during oscillations of interest

To visualize FR modulation by fast network oscillations (Fig. 3*A*, Extended Data Fig. 3-1), spikes of individual cells were aligned to the power peaks of oscillatory events of interest, and FRs were calculated using 10-ms bins for SWRs, HFOs, and cRipples and in 50-ms bins for spindles. The peak-aligned FRs were smoothed with a Gaussian kernel (*σ* = 50 ms for SWRs, HFOs, and cRipples and 250 ms for spindles), after which event-averaged FRs were obtained for each neuron.

FR gain during oscillations of interest was analyzed by computing FRs within and outside each type of oscillations in NREM sleep, including sleep SWRs, HFOs, cRipples, and spindles. The FR within oscillatory events was calculated as the number of spikes occurring within the events divided by the total duration of those events, while the FR outside oscillatory events was determined using the same method for periods outside the events. FR gain during oscillations was then defined as the ratio of FR within oscillatory events to FR outside oscillatory events. For awake SWRs, FR gain was computed as the ratio of FR during awake SWRs to FR during non-SWR periods within awake epochs.

### Duration and occurrence rate of oscillatory events

The mean duration of oscillatory events in NREM sleep (sleep SWRs, HFOs, cRipples, and spindles) was measured as the average duration of individual events detected within NREM epochs. Similarly, the mean duration of awake SWRs was calculated as the mean duration of individual SWRs detected during awake periods. The occurrence rate of oscillatory events in NREM sleep was determined by dividing the number of detected events by the total duration of NREM epochs. For awake SWRs, the occurrence rate was calculated by dividing the number of awake SWRs by the total duration of awake epochs.

### Coactivity *Z*-score

The degree of coactivation during oscillations of interest for a given pair of cells was quantified using the coactivity *Z*-score (Cheng and Frank, 2008). Assuming that two neurons *A* and *B* were active (emitted at least one spike during an event) in *n_A_* and *n_B_* events, respectively, out of a total of *N* oscillatory events, the raw coactivity score *R* was defined as the number of oscillatory events in which both neurons were active. Since *R* follows a hypergeometric distribution when the two neurons are independently active (Sheskin, 2004; Fig. 4*A*, Extended Data Fig. 4-1), the expected value of *R*, *E*, and the variance of *R*, *σ*^2^, were calculated as follows:

$$E=\frac{n_{A}n_{B}}{N},$$

$$\;\sigma^{2}=\;\frac{n_{A}n_{B}(N-n_{A})(N-n_{B})}{N^{2}(N-1)}.$$

The coactivity *Z-*score (*Z*) was then defined as the normalized *R* with *E* and *σ*:

$$\;Z\;=\frac{R-E\;}{\sigma \;}.$$

### NREM-preferring neuron response indices for SWRs, HFOs, cRipples, and spindles

NREM-preferring neuron response indices for oscillations of interest (SWRs, HFOs, cRipples, and spindles) during NREM sleep were calculated following the approach described by Harvey et al. (2023). For each *i*th oscillatory event, the number of active neurons—defined as neurons that fired at least once during the event—was counted separately for REM- and NREM-preferring neurons within the brain region where the oscillation was detected. These counts were denoted as *N_R_*(*i*) and *N_N_*(*i*), respectively. The fraction of active NREM-preferring neurons among all active neurons, *X*(*i*), was then computed as follows:

$$X(i)=\frac{N_{N}(i)}{N_{N}(i)+N_{R}(i)}.$$

Additionally, the FR of each neuron in the target region during the *i*th oscillatory event was calculated and denoted as *Y*(*i*). This process was repeated across all oscillatory events (*i* = 1, 2, 3, …, total number of events). For each neuron, the NREM-preferring neuron response index was defined as the Pearson's correlation coefficient between *X* and *Y*. These indices were computed separately for each neuron in the target regions for SWRs, HFOs, cRipples, and spindles.

### FR gain during shock presentation

For each cell, the maximum FR during the 2-s shock presentations was obtained from the peak of the peri-stimulus time histogram, which was calculated using a bin width of 0.1 s and averaged across shock presentations. The FR gain during shock presentation was then computed by dividing the maximum FR during the shock by the mean FR during the 20 s preceding the first CS.

### Statistical analyses

Statistical analyses were performed using the NumPy and SciPy modules in Python (Harris et al., 2020; Virtanen et al., 2020; http://numpy.org/, http://www.scipy.org/) and EZR (Kanda, 2013; http://www.jichi.ac.jp/saitama-sct/SaitamaHP.files/statmed.html). Comparisons of FRs, FR gains during oscillations, and NREM-preferring neuron response indices between NREM- and REM-preferring neurons were performed using the Mann–Whitney *U* test. FRs during the first and last NREM epochs were compared using the Wilcoxon signed-rank test. To compare coactivity *Z-*scores of cell pairs, the post hoc Steel–Dwass test was applied when the *p* value of the Kruskal–Wallis test was <0.05. Data in bar graphs are presented as mean ± standard error of mean (SEM). All *p* values indicate Spearman's rank-order correlation coefficient. See Extended Data for details of individual analyses.

### Code accessibility

The analysis was performed using Python 3.9.5, running on a Windows 10 system equipped with an Intel Core i7-10870H CPU. The code generated for the analyses in this paper is available at https://github.com/satonisaku/Risa-Kajiya-2025.

### Data availability

The data used in this study are available from the corresponding authors upon request.

## Results

### Sleep-state modulation of firing activity varied across neurons

The effect of sleep state on neuronal firing was examined using previously obtained 17-h continuous recordings of single-unit activity (143, 479, and 232 neurons recorded in the vCA1, PL5, and BLA, respectively, from 15 rats in total; Table 1, Extended Data Table 1-1) along with LFPs from fear-conditioned rats (Miyawaki and Mizuseki, 2022; Fig. 1*A*). In dorsal CA1, neuronal FRs exhibit a sawtooth pattern across NREM–REM–NREM cycles, with modest increases during NREM followed by greater decreases during REM, resulting in an overall decline in FR across sleep (Grosmark et al., 2012; Miyawaki and Diba, 2016). However, how these patterns vary across individual neurons remains incompletely understood. To investigate this, FRs were analyzed across NREM–REM–NREM triplets (Extended Data Figs. 1-1, 1-2; see Materials and Methods), revealing that REM/NREM preference varied substantially across neurons (Fig. 1*B–D*). Each neuron's sleep-state preference was quantified using a REM-preference index, calculated using all NREM and REM epochs lasting >50 s (see Materials and Methods). The index ranges from −1 (exclusive NREM firing) to 1 (exclusive REM firing), with values near 0 indicating no sleep-state modulation. To determine whether a neuron's REM-preference index significantly deviated from 0, the actual value was compared with a null distribution generated by time bin label shuffling for each neuron (Fig. 1*B*, middle column). The analysis revealed that >50% of excitatory neurons exhibited significantly higher firing during REM than during NREM sleep. A smaller fraction of excitatory neurons displayed a preference for NREM sleep (Fig. 1*E*,*F*; Table 1). Specifically, 63.0, 72.5, and 76.1% of excitatory neurons in vCA1, PL5, and BLA, respectively, were REM preferring, while 30.4, 20.3, and 19.6% were NREM preferring. Putative inhibitory neurons showed qualitatively similar results, with 100.0, 70.0, and 83.3% classified as REM-preferring and 0.0, 28.0, and 16.7% as NREM-preferring in vCA1, PL5, and BLA, respectively (Fig. 1*E*,*F*; Table 1). The robustness of this classification was confirmed by testing more stringent thresholds, which did not qualitatively alter the proportion of REM-/NREM-preferring neurons (Extended Data Fig. 1-3). Due to the small number of NREM-preferring inhibitory neurons, further analyses focused on excitatory neurons unless otherwise noted. The REM-preference indices of individual neurons remained significantly correlated across different hc sessions, suggesting that REM/NREM preference is a stable property of individual neurons (Fig. 1*G*,*H*; Extended Data Figs. 1-4, 1-5). Bootstrap analysis further confirmed the robustness of these correlations, even when accounting for variation in the number of recorded neurons (Extended Data Figs. 1-6, 1-7). These findings demonstrate that while most excitatory neurons preferentially fire during REM sleep, a substantial minority—ranging from one-fifth to one-third—preferentially fire during NREM sleep.

**Table 1.** Number of NREM-preferring, REM-preferring, and non-significant neurons

	Excitatory neurons			Inhibitory neurons		
	vCA1	PL5	BLA	vCA1	PL5	BLA
NREM-preferring neurons	28 (28, 28, 28)	85 (85, 85, 85)	41 (39, 41, 41)	0	14	3
REM-preferring neurons	58 (58, 58, 58)	303 (303, 303, 303)	159 (144, 159, 159)	46	35	15
Non-significant neurons	6 (6, 6, 6)	30 (30, 30, 30)	9 (8, 9, 9)	0	1	0
Total	92 (92, 92, 92)	418 (418, 418, 418)	209 (191, 209, 209)	46	50	18
Examined rats	8 (8, 8, 8)	8 (8, 8, 8)	12 (11, 12, 12)	7	8	7

Number of NREM- and REM-preferring and non-significant neurons among excitatory and inhibitory neurons in the vCA1, PL5, and BLA. The bottom row indicates the number of rats from which neurons in each brain region were recorded. Numbers in parentheses in the upper four rows indicate the number of excitatory neurons recorded simultaneously with SWRs in the ventral hippocampus, cortical ripples (cRipples) and spindles in the prelimbic cortex, and high-frequency oscillations (HFOs) in the amygdala. Numbers in parentheses in the bottom row indicate the number of rats from which excitatory neurons were recorded simultaneously with SWRs in the ventral hippocampus, cRipples and spindles in the prelimbic cortex, and HFOs in the amygdala. BLA excitatory neurons recorded from one rat in which hippocampal SWRs could not be detected were excluded from analyses requiring SWR detection.

10.1523/ENEURO.0575-24.2025.t1-1 Table 1-1 **Classification of excitatory and inhibitory neurons** **(A)** Example cross-correlograms (CCGs) of spike times from neuron pairs showing significant spike transmission (left) and suppression (right). Red and cyan lines indicate the 99% confidence intervals (CIs) for detected CCG peaks and troughs within the [–5, + 5] ms range, respectively. The gray band represents the 99% CI at each time point, obtained by jittering spike timings. The orange background highlights the [+1, + 4] ms period used to evaluate the significance of peaks and troughs. **(B)** Mean waveforms of all recorded neurons in each brain region (top), and scatter plots of spike width versus mean firing rate (bottom). The vertical scales of the mean waveforms are normalized by spike amplitudes. Colors represent cell types: red for putative excitatory neurons, blue for putative inhibitory neurons, and gray for non-classified neurons. (A) and (B): Reproduced from Miyawaki and Mizuseki (2022) under a CC-BY 4.0 license. Download Table 1-1, TIF file.

10.1523/ENEURO.0575-24.2025.f1-1 Figure 1-1 **Duration of NREM and REM sleep and wakefulness in the home cage (hc) and behavioral sessions** Mean ± standard deviation of the duration and proportion of NREM sleep, REM sleep, and wakefulness during each home cage (hc) and behavioral session (n = 15 rats). Download Figure 1-1, DOCX file.

**Figure 2.**
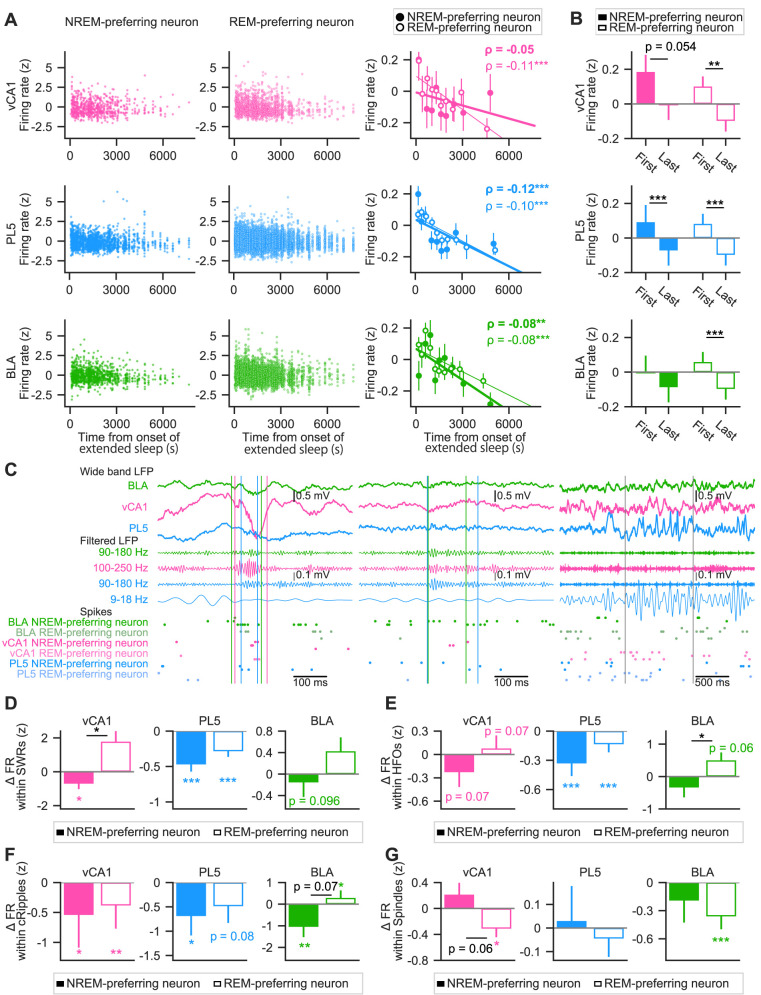
Firing rate changes of NREM- and REM-preferring excitatory neurons across sleep. ***A***, Mean firing rates (FRs) of excitatory neurons in the vCA1 (top row), PL5 (middle row), and BLA (bottom row) decreased over the course of sleep. Left and middle columns, *Z*-scored mean FRs during NREM epochs of extended sleep are plotted against time from sleep onset for NREM-preferring (left) and REM-preferring (middle) neurons. Each circle (filled for NREM-preferring, hollow for REM-preferring) represents the mean FR of an individual neuron during a single NREM epoch; the *x*-axis indicates the time from the onset of extended sleep to the midpoint of each epoch. Right column: *Z*-scored mean FRs across NREM epochs, grouped into 10 equally sized time bins. Regression lines (thick for NREM-preferring, thin for REM-preferring) were fitted to the data shown in the left and middle panels, respectively. Spearman's rank-order correlation coefficient (*ρ*) is displayed in the top right corner of each plot (bold for NREM-preferring, regular font for REM-preferring). **p < 0.01, ***p < 0.001. Error bars represent the standard error of the mean (SEM). ***B***, *Z-*scored mean FRs during the first and last NREM epochs of extended sleep for vCA1 (top), PL5 (middle), and BLA (bottom). NREM-preferring (solid bars) and REM-preferring (hollow bars) neurons are shown separately. Error bars represent SEM. **p < 0.01, ***p < 0.001; Wilcoxon signed-rank test. ***C***, Representative examples of SWRs, HFOs, cRipples, and spindles recorded from a rat. Top, Wideband and filtered local field potential (LFP) traces. Bottom, Spike timings of representative NREM-preferring (vivid colors) and REM-preferring (pale colors) neurons. Trace and dot colors denote brain regions (green for BLA, pink for vCA1, and light blue for PL5). Vertical lines indicate the onset and offset of each oscillatory event (green for HFOs, magenta for SWRs, cyan for cRipples, and gray for spindles). Note that the time window of the rightmost example is five times longer than those of the others. ***D–G***, Comparison of FR changes during SWRs (***D***), HFOs (***E***), cRipples (***F***), or spindles (***G***) across sleep between NREM-preferring (filled bars) and REM-preferring (hollow bars) neurons. Mean FR changes between the first and last NREM epochs of extended sleep are shown. Error bars indicate SEM. *p < 0.05, **p < 0.01, ***p < 0.0011; Wilcoxon signed-rank test for within-group comparisons (colored) and Mann–Whitney *U* test for between-group comparisons (black). Details of the statistical tests are provided in Extended Data Figure 2-1. The durations and occurrence rates of network oscillatory events are summarized in Extended Data Figure 2-2.

10.1523/ENEURO.0575-24.2025.f1-2 Figure 1-2 **Number of NREM–REM–NREM triplets in extended sleep** Total number of NREM–REM–NREM triplets and rats analyzed, related to Fig. 1B and D. Download Figure 1-2, DOCX file.

**Figure 3.**
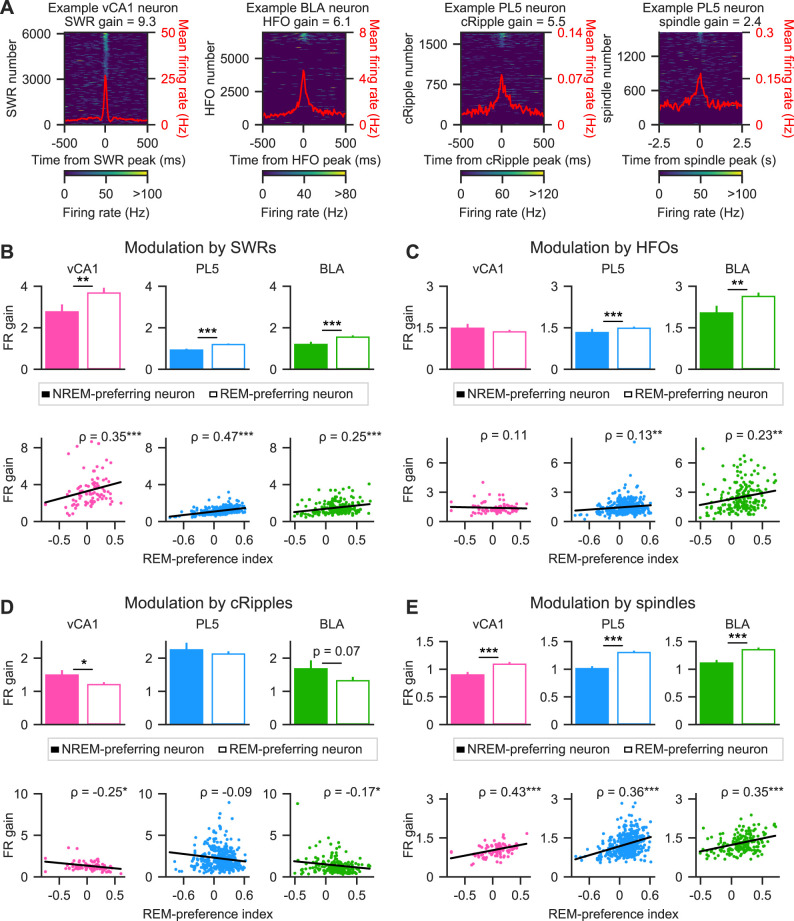
Modulation of NREM- and REM-preferring excitatory neuron activity by fast network oscillations during sleep. ***A***, Firing rates (FRs) of representative neurons aligned to the power peaks of SWRs, HFOs, cRipples, or spindles. Perievent FR aligned with the power peak of each oscillatory event is color coded and sorted by FR peak. The red line represents the mean FR across oscillatory events. The FR gain during the corresponding oscillation events is indicated at the top of each panel. Additional representative examples are shown in Extended Data Figure 3-1. ***B–E***, FR gains during SWRs (***B***), HFOs (***C***), cRipples (***D***), and cortical spindles (***E***). Top panels, Mean FR gains of NREM-preferring (solid bars) and REM-preferring (hollow bars) neurons. Error bars represent the standard error of the mean (SEM). Bottom panels, Relationship between REM-preference indices and FR gains. Each dot represents a single neuron. All neurons—NREM-preferring, REM-preferring, and non-significant—are included in the plots, except for a single outlier in the middle and right bottom panels of ***D***, which is beyond the plotted *y*-axis range. Nevertheless, all data points, including outliers, were included in the statistical analyses. Linear regression lines are shown in black, and Spearman's rank-order correlation coefficient (*ρ*) is presented in the top right of each panel. *p < 0.05, **p < 0.01, ***p < 0.001; Mann–Whitney *U* test was used for group comparisons in the top panels, and significance of Spearman's rank-order correlation was used for statistical testing in the bottom panels. The number of neurons included in each analysis is summarized in Table 1, and full statistical details are provided in Extended Data Figure 3-2.

10.1523/ENEURO.0575-24.2025.f1-3 Figure 1-3 **Comparison of the number of NREM- and REM-preferring neurons and non-significant neurons defined by different classification thresholds** To classify neurons as REM-preferring, NREM-preferring, or non-significant neurons, we generated a distribution of REM-preference indices from shuffled surrogates (REM/NREM bin label shuffling, *n* = 1,000) for each neuron (see *Classification of REM- and NREM-preferring neurons* in *Materials and Methods*). Throughout the paper, a neuron was labeled as REM- or NREM-preferring if its actual REM-preference index exceeded the 97.5th percentile or fell below the 2.5th percentile of the surrogate distribution, respectively (*p* < 0.05, 5% criterion). Otherwise, it was labeled as non-significant. Here, more stringent thresholds were tested for classifying REM- and NREM-preferring neurons (*p* < 0.01, 1% criterion; and *p* < 0.002, 0.2% criterion). Percentages in parentheses indicate the proportion of neurons initially classified using 5% criterion that also met the more stringent criterion. Download Figure 1-3, DOCX file.

**Figure 4.**
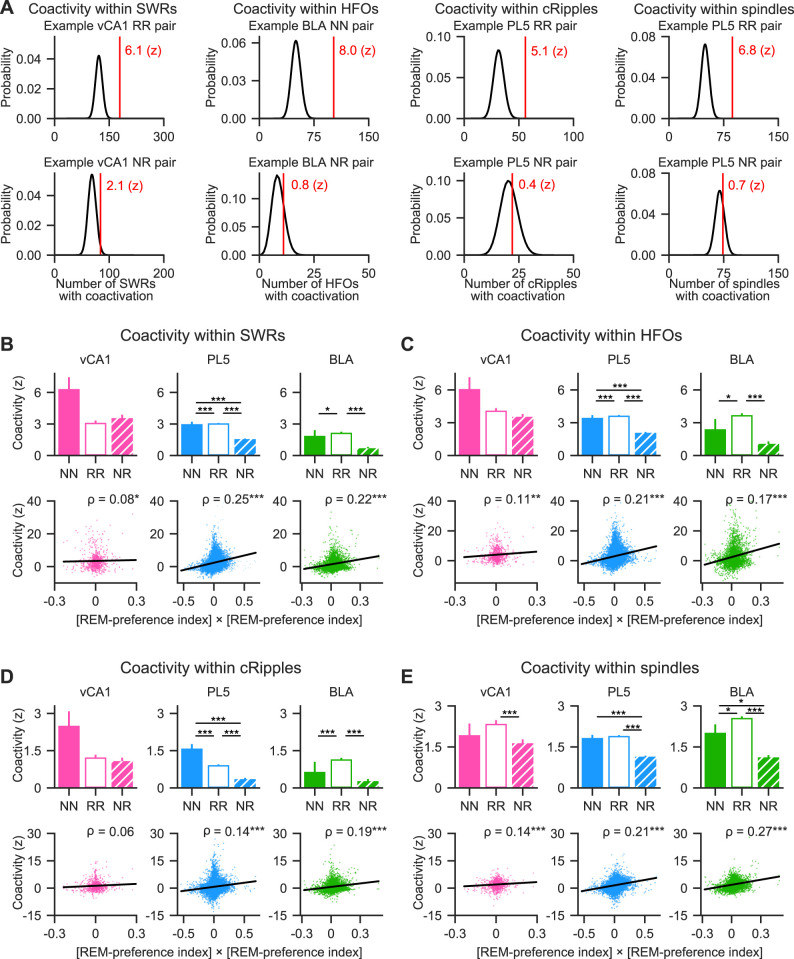
Within-regional coactivity of excitatory neuron pairs during fast network oscillations in sleep. ***A***, Coactivation of example neuron pairs during SWRs, HFOs, cRipples, or spindles. The black curve represents the theoretical distribution of coactivation event numbers under the null hypothesis of independent firing (see Materials and Methods for details). The observed number of coactivation events is indicated by the red vertical line, and the corresponding coactivity *Z*-score is displayed next to the line. Example neuron pairs with similar or opposing sleep-state preferences are shown in top and bottom plots, respectively. For more examples, see Extended Data Figure 4-1. ***B–E***, Coactivity *Z*-scores for SWRs (***B***), HFOs (***C***), cRipples (***D***), and spindles (***E***), computed for excitatory neuron pairs within the vCA1 (pink, left), PL5 (light blue, middle), and BLA (green, right). Top panels, Mean coactivity *Z*-scores are shown for each neuron pair type. Error bars represent the standard error of the mean (SEM). Bottom panels, Relationship between the product of REM-preference indices of each neuron pair and corresponding coactivity *Z*-score. All neuron pairs—including NN, RR, NR, and pairs containing non-significant neurons—are shown. Each dot represents a single neuron pair. Linear regression lines are shown in black, and Spearman's rank-order correlation coefficient (*ρ*) is indicated in the top right of each panel. *p < 0.05, **p < 0.01, ***p < 0.001; post hoc Steel–Dwass test following the Kruskal–Wallis test for the top panels and significance of the Spearman's rank-order correlation for the bottom panels. Pairs containing non-significant neurons were included in the correlation analyses. The number of neuron pairs analyzed and details of the statistical tests are provided in Extended Data Figure 4-2. NN, NREM-preferring neuron pairs; RR, REM-preferring neuron pairs; NR, pairs of NREM- and REM-preferring neurons.

10.1523/ENEURO.0575-24.2025.f1-4 Figure 1-4 **Comparison of REM-preference indices of excitatory neurons across home cage sessions** Same as Fig. 1G, but excitatory neurons in the vCA1, PL5, and BLA are shown separately. REM-preference indices of excitatory neurons were calculated within each home cage (hc) session. REM/NREM preferences remained largely stable across sessions. Dot colors represent neuron types classified based on all concatenated sleep epochs, as in Fig. 1G: black circles indicate NREM-preferring neurons, gray circles indicate REM-preferring neurons, and orange triangles indicate non-significant neurons. Statistical details are provided in Extended Data Fig. 1-5. Download Figure 1-4, TIF file.

**Figure 5.**
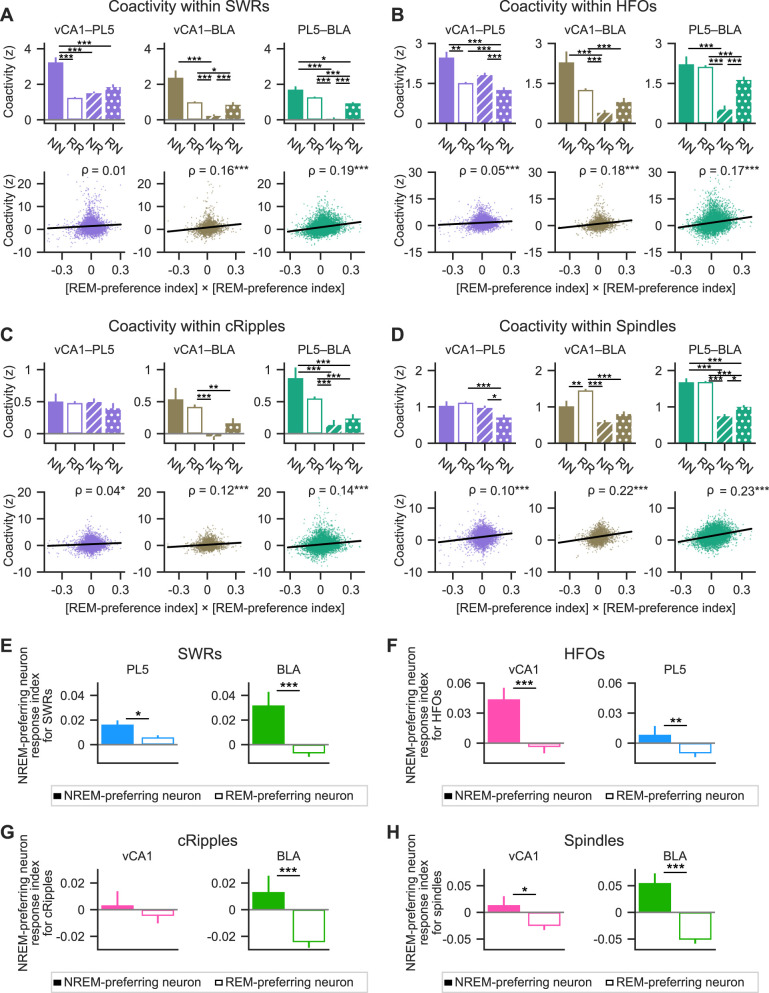
Within-regional coactivity of excitatory neuron pairs during fast network oscillations in sleep. ***A–D***, Cross-regional coactivity *Z*-scores for (***A***) SWRs, (***B***) HFOs, (***C***) cRipples, and (***D***) cortical spindles for neuron pairs spanning vCA1–PL5 (left), vCA1–BLA (middle), and PL5–BLA (right). Data are shown separately for each pair type. Top panels, Mean coactivity *Z*-scores for each pair type. Error bars represent the standard error of the mean (SEM). Bottom panels, Relationship between the product of REM-preference indices and coactivity *Z*-score for each neuron pair. Each dot represents a single neuron pair. All pair types—including those containing non-significant neurons—are included. Black lines represent linear regressions; Spearman's rank-order correlation coefficient (*ρ*) is shown in the top right corner of each plot. *p < 0.05, **p < 0.01, ***p < 0.001; top panels, post hoc Steel–Dwass test following Kruskal–Wallis test; bottom panels, significance of Spearman's rank-order correlation. ***E–H***, NREM-preferring neuron response indices for SWRs (***E***), HFOs (***F***), cRipples (***G***), and cortical spindles (***H***). Solid bars indicate NREM-preferring neurons; hollow bars indicate REM-preferring neurons. Error bars represent SEM. *p < 0.05, **p < 0.01, ***p < 0.001; Mann–Whitney *U* test. Details of all statistical analyses are provided in Extended Data Figure 5-1. NN, NREM-preferring neuron pairs; RR, REM-preferring neuron pairs; NR and RN, pairs of NREM- and REM-preferring neurons, where the order of “*N*” and “*R*” corresponds to the order of the brain regions of the constituent neurons.

10.1523/ENEURO.0575-24.2025.f1-5 Figure 1-5 **Comparison of REM-preference indices between home cage sessions** Spearman’s rank-order correlation coefficients of REM-preference indices between home cage sessions in the vCA1, PL5, and BLA, corresponding to Fig. 1H and Extended Data Fig 1-4. Download Figure 1-5, DOCX file.

**Figure 6.**
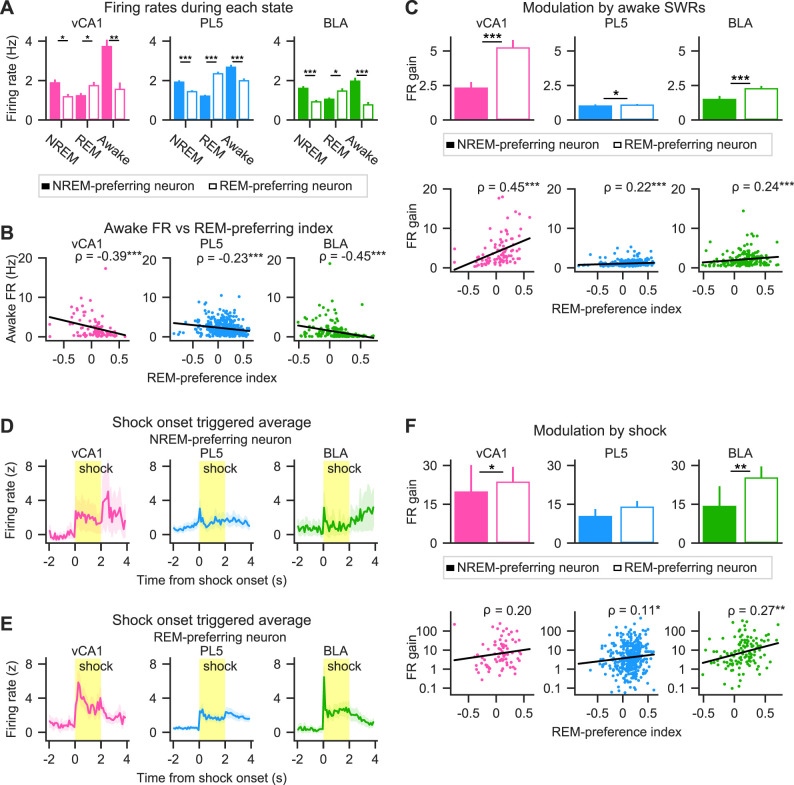
Activity of NREM- and REM-preferring excitatory neurons during wakefulness. ***A***, Mean firing rates (FRs) across behavioral states. During wakefulness, NREM-preferring neurons (filled bars) showed significantly higher FRs than REM-preferring neurons (hollow bars). Error bars represent standard error of the mean (SEM). *p < 0.05, **p < 0.01, ***p < 0.001; Mann–Whitney *U* test. ***B***, Relationship between FRs during wakefulness and REM-preference indices. Each dot represents a single neuron. All neurons—including NREM-preferring, REM-preferring, and non-significant—are plotted and included in the Spearman's rank-order correlation analysis. ***p < 0.001. ***C***, REM-preferring neurons exhibited greater FR gains during awake SWRs than NREM-preferring neurons. Top, FR gains during awake SWRs. Error bars represent SEM. Bottom, Relationship between FR gains during awake SWRs and REM-preference indices. Each dot represents a single neuron; all neurons are plotted and included in the Spearman's rank-order correlation analysis. *p < 0.05, ***p < 0.001; Mann–Whitney *U* test (top), significance of Spearman's rank-order correlation (bottom). ***D***, ***E***, Shock onset-triggered averages of FRs for NREM-preferring (***D***) and REM-preferring (***E***) neurons. Solid lines indicate mean FRs; shaded areas represent SEM. Yellow background highlights the shock delivery period. ***F***, REM-preferring neurons in vCA1 and BLA displayed larger increases in firing during shock presentation compared with NREM-preferring neurons. Top, Mean FR gains during shock for NREM-preferring (filled bars) and REM-preferring (hollow bars) neurons. Error bars indicate SEM. Bottom, Relationship between FR gains during shocks and REM-preference indices. Each dot represents a single neuron; all neurons are plotted and included in the Spearman's rank-order correlation analysis. *p < 0.05, **p < 0.01; Mann–Whitney *U* test (top), Spearman's rank-order correlation (bottom). The number of neurons included in each analysis is summarized in Table 1. Statistical test details are provided in Extended Data Figure 6-1.

10.1523/ENEURO.0575-24.2025.f1-6 Figure 1-6 **Correlation of REM-preference indices across home cage sessions using bootstrapping** Same as Fig. 1H, but Spearman’s rank-order correlation coefficients of REM-preference indices between home cage sessions in the vCA1, PL5, and BLA were estimated using a bootstrapping method. An equal number of neurons (n = 92) was sampled in each brain region to control for differences in cell counts. Medians of the bootstrapped rank-order correlation coefficients are shown. *p < 0.05, **p < 0.01, ***p < 0.001. See Extended Data Fig.1-7 for statistical details. Download Figure 1-6, TIF file.

**Figure 7.**
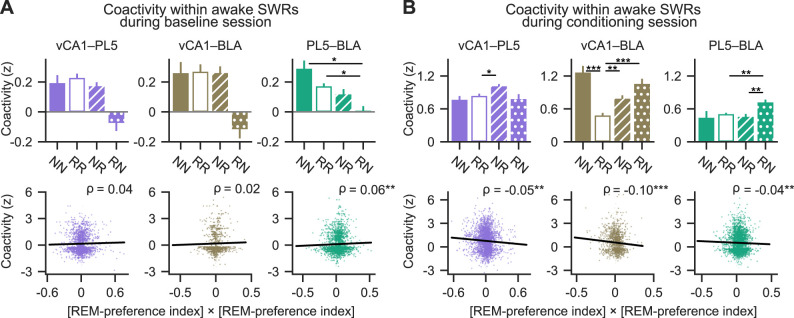
Inter-regional coactivity of excitatory neuron pairs during awake SWRs. Cross-regional coactivity during awake SWRs during the baseline (***A***) and conditioning (***B***) sessions for neuron pairs spanning vCA1–PL5 (left), vCA1–BLA (middle), and PL5–BLA (right). Top panels, Mean coactivity *Z*-scores for each neuron pair type (NN, RR, NR, and RN pairs). Error bars represent the standard error of the mean (SEM). Bottom panels, Relationship between the products of REM-preference indices and coactivity *Z*-scores. Each dot represents a single neuron pair, including NN, RR, NR, RN, and pairs containing non-significant neurons. Black lines indicate linear regression fits, and Spearman's rank-order correlation coefficients (*ρ*) are shown in the top right corner of each plot. *p < 0.05, **p < 0.01, ***p < 0.001; post hoc Steel–Dwass test following Kruskal–Wallis test (top panels) and significance of Spearman's rank-order correlation (bottom panels). Pairs with non-significant neurons were included in the correlation analyses. Details of the statistical tests are provided in Extended Data Figure 7-3.

10.1523/ENEURO.0575-24.2025.f1-7 Figure 1-7 **Spearman’s rank-order correlation coefficients between REM-preference indices of different home cage sessions evaluated using bootstrapping** Statistical details for Extended Data Fig. 1-6. As in Extended Data Fig. 1-5, this table presents Spearman’s rank-order correlation coefficients (*ρ*) of REM-preference indices between different home cage sessions, estimated using bootstrapping. The median correlation coefficient and the *p*-value against the null hypothesis (that the correlation coefficient is zero) are reported. Download Figure 1-7, DOCX file.

**Figure 8.**
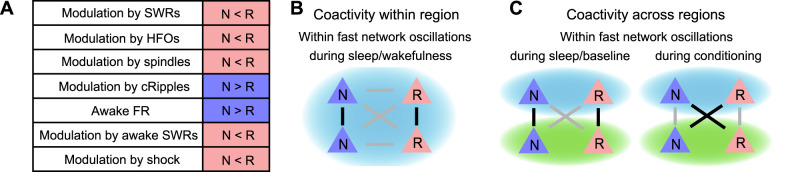
Summary of differences between NREM-preferring and REM-preferring neurons. ***A***, Activity modulation of NREM-preferring and REM-preferring neurons. REM-preferring neurons exhibited stronger activation during fast network oscillations (excluding cRipples) and responded more robustly to shock stimuli (highlighted with a pink background). In contrast, NREM-preferring neurons were more strongly activated by cRipples and showed higher average firing rates during wakefulness (violet background). ***B***, Within-regional coactivity among NREM-preferring and REM-preferring neurons. NREM-preferring and REM-preferring neurons are illustrated as violet and pink triangles, respectively. Coactivity strength is indicated by the intensity of the connecting lines. During fast network oscillations in NREM sleep and during awake SWRs in both baseline and conditioning sessions, coactivity *Z*-scores positively correlated with the products of REM-preference indices in at least one brain region, with no significant negative correlations in any regions examined. These findings indicate stronger coactivation between neuron pairs with similar REM/NREM preferences (black lines) compared with those with differing preferences (gray lines). ***C***, Cross-regional coactivity among NREM- and REM-preferring neurons. Neurons with the same or different background colors belong to the same or different brain regions, respectively. During fast network oscillations in NREM sleep and awake SWRs in the baseline session, coactivity *Z*-scores again positively correlated with the products of REM-preference indices in at least one brain region pair, with no significant negative correlations in any region pairs examined. In contrast, during awake SWRs in the conditioning session, correlations were significantly negative across all region pairs, indicating that neurons with differing REM/NREM preferences exhibited stronger coactivation than similarly tuned pairs. N and R represent NREM-preferring and REM-preferring neurons, respectively, in all panels.

10.1523/ENEURO.0575-24.2025.f1-8 Figure 1-8 **Comparison of Spearman’s rank-order correlation of REM-preference indices estimated via bootstrapping** **(A)** For each brain region, an equal number of neurons (n = 92) were resampled with replacement, and Spearman’s rank-order correlation coefficients of REM-preference indices were calculated between temporally adjacent home cage (hc) sessions. The difference in correlation coefficients between hc session pairs was then computed. This procedure was repeated 5,000 and the resulting distributions with their medians (orange bars) are shown. *p < 0.05. See Extended Data Fig. 1-9 for statistical details. **(B)** For each pair of brain regions, 92 neurons were resampled with replacement per region. Spearman’s rank-order correlation coefficients of REM-preference indices between temporally adjacent home cage sessions were computed for each region, and inter-region differences were calculated. This procedure was also repeated 5,000 times and the resulting distributions with their medians (orange bars) are shown. *p < 0.05, **p < 0.01, ***p < 0.001. See Extended Data Fig. 1-9 for statistical details. Spearman’s rank-order correlation coefficient between hc*X* and hc*Y* is denoted as *ρ_XY_*. Download Figure 1-8, TIF file.

10.1523/ENEURO.0575-24.2025.f1-9 Figure 1-9 **Comparison of Spearman’s rank-order correlation coefficients of REM-preference indices** Statistical details for Extended Data Fig. 1-8. This table compares Spearman’s rank-order correlation coefficients (*ρ*) for REM-preference indices between temporally adjacent home cage (hc) sessions. **Top:** Statistical details for Extended Data Fig. 1-8A. Comparison of correlation coefficients between consecutive hc session pairs within each brain region. **Bottom:** Statistical details for Extended Data Fig. 1-8B. Comparison of correlation coefficients between brain regions. Bootstrapping was used to estimate the 95% confidence intervals (CIs) of the differences in correlation coefficients for both comparisons. The Spearman’s correlation coefficient of REM-preference indices between sessions hc*X* and hc*Y* is denoted as *ρ*_XY_. Download Figure 1-9, DOCX file.

The stability of REM preference was further examined with respect to interleaving experiences between hc sessions. Spearman's rank-order correlation coefficients of REM-preference indices were computed using a bootstrapped dataset with an equal number of neurons resampled across brain regions to control for differences in cell number. No significant differences in correlation coefficients were observed between temporally adjacent hc session pairs in any of the brain regions examined, except for the difference between the correlation coefficient of hc2 versus hc3 and that of hc3 versus hc4 in the BLA (Extended Data Figs. 1-8*A*, 1-9). These results indicate that the stability of REM preference remained largely unaffected by intervening experiences under the conditions of this study.

Finally, the stability of REM preference was assessed across brain regions. Correlations of REM-preference indices between temporally adjacent hc sessions were found to be stronger in PL5 and BLA than in vCA1 (Extended Data Figs. 1-8*B*, 1-9), suggesting that REM preference is more stable in PL5 and BLA than in vCA1. The relatively dynamic nature of REM preference in vCA1 may reflect the region's involvement in learning and memory processes.

### REM- and NREM-preferring neurons exhibited similar decreases in FR but distinct firing modulation within fast network oscillations during sleep

The synaptic homeostasis hypothesis posits that synaptic strength weakens during sleep, leading to a net decrease in neuronal firing activity (Tononi and Cirelli, 2003, 2014). Supporting this hypothesis, prior studies have reported sleep-associated decreases in FRs in the dorsal hippocampus (Grosmark et al., 2012; Miyawaki and Diba, 2016) and neocortex (Vyazovskiy et al., 2009; Watson et al., 2016; Torrado Pacheco et al., 2021). However, whether REM- and NREM-preferring neurons exhibit similar patterns of activity change remains unclear. To address this question, changes in FRs were examined across sleep, focusing on “extended sleep”—periods lasting >30 min without interruptions of >60 s (Miyawaki and Diba, 2016; Torrado Pacheco et al., 2021). Excitatory neurons were analyzed separately in each brain region. Consistent with prior findings in the barrel cortex and dorsal CA1 (Vyazovskiy et al., 2009; Miyawaki and Diba, 2016), a gradual decrease in mean FRs was observed during NREM epochs in vCA1, PL5, and BLA. *Z*-scored mean FRs of individual neurons showed negative correlations with time from sleep onset (vCA1, *ρ* = −0.09, *p* = 2.4 × 10^−5^; PL5, *ρ* = −0.11, *p* = 5.6 × 10^−26^; BLA, *ρ* = −0.08, *p* = 5.9 × 10^−9^) and significantly declined from the first to last NREM epoch in extended sleep (ΔFR [*z*] = −0.19 ± 0.05, −0.18 ± 0.02, and −0.15 ± 0.04 for vCA1, PL5, and BLA, respectively; *p* = 1.0 × 10^−4^, 2.8 × 10^−17^, and 9.2 × 10^−6^; Wilcoxon signed-rank test; mean ± SEM). When analyzed by sleep preference, both REM- and NREM-preferring neurons showed significant reductions in FR during sleep across all regions, except for NREM-preferring neurons in vCA1 (Fig. 2*A*). REM-preferring neurons exhibited significant FR decreases from the first to last NREM epoch across all brain regions (Fig. 2*B*). NREM-preferring neurons also exhibited decreasing trends, though statistical significance for the reduction was observed only in the PL5 (Fig. 2*B*). These results suggest that both REM- and NREM-preferring neurons undergo FR reductions during sleep, though the patterns are region-specific and influenced by sleep preference.

10.1523/ENEURO.0575-24.2025.f2-1 Figure 2-1 **Statistical details for Fig. 2** Number of neurons analyzed during extended sleep and the statistical tests used in Fig. 2. *N* and *R* denote NREM- and REM-preferring neurons, respectively. **(A)** The firing rate (FR) of each neuron was calculated for every NREM epoch during extended sleep. Each NREM epoch in which each cell’s FR was calculated is referred to as a “cell-epoch.” The total number of cell-epochs summed across all neurons and the total number of neurons used in these calculations are also reported. n.a., not applicable. **(B)** The FR of each neuron was calculated during the first and last NREM epoch of each extended sleep period. Each extended sleep period in which these values were computed for each cell is referred to as a “cell-period.” The total number of cell-periods summed across all neurons and the total number of neurons used in this analysis are provided. **(D–G)** The change in FR within fast network oscillations during the first and last NREM epochs was calculated for each cell in each extended sleep period. As in (B), each extended sleep period for which these values were calculated for each cell is referred to as a “cell-period”. In addition to the total number of cell-periods summed across neurons, the total number of neurons used in this analysis is also reported. Download Figure 2-1, XLS file.

10.1523/ENEURO.0575-24.2025.f2-2 Figure 2-2 **Duration and occurrence rate of network oscillatory events** The duration (median with 1st and 3rd quartiles) and occurrence rate (mean ± standard deviation) of fast network oscillations detected in vCA1 (sleep and awake SWRs), PL5 (cRipples and spindles), and BLA (HFOs) recordings, and the number of rats in which the respective oscillatory events were examined. Download Figure 2-2, DOCX file.

In the dorsal hippocampus, FRs within SWRs have been reported to increase during sleep, despite an overall reduction in baseline FRs (Grosmark et al., 2012). However, whether this enhancement occurs uniformly across neuronal subtypes—such as REM- and NREM-preferring neurons—and across different brain regions remains unclear. To investigate this, changes in neuronal activity during fast network oscillations (Fig. 2*C*) were assessed across sleep. Specifically, changes in within-oscillatory event FRs were quantified by subtracting the *Z*-scored mean FRs during the first NREM epoch from those during the last NREM epoch of extended sleep. In the vCA1, within-SWR firing of REM-preferring neurons showed an increasing trend, though not statistically significant, whereas that of NREM-preferring neurons exhibited a significant decrease. Consequently, changes in within-SWR FRs significantly differed between REM- and NREM-preferring neurons in this region (Fig. 2*D*). In contrast, within-SWR firing significantly decreased across sleep for both neuron types in the PL5 (Fig. 2*D*). In the BLA, NREM-preferring neurons showed a decreasing trend, while REM-preferring neurons exhibited no significant change (Fig. 2*D*).

The same analysis was extended to HFOs, cRipples, and spindles to examine how FRs within these oscillations change during extended sleep. Changes in firing within amygdalar HFOs (Fig. 2*E*) were similar to those within SWRs. Specifically, within-HFO firing showed increasing trends in REM-preferring neurons, while it tended to decrease in NREM-preferring neurons in the BLA (Fig. 2*E*). Consequently, the changes in within-HFO FRs of REM- and NREM-preferring neurons across extended sleep were statistically different in the BLA (Fig. 2*E*). Similar trends were observed for vCA1 neurons; NREM- and REM-preferring neurons showed a trend of decreasing and increasing within-HFO firing, respectively (Fig. 2*E*). In the PL5, the within-HFO firing of both NREM- and REM-preferring neurons showed a significant decrease across sleep (Fig. 2*E*). For cRipples, within-event FRs were reduced in most cell types. Both NREM- and REM-preferring neurons in the vCA1 significantly reduced within-cRipple firing (Fig. 2*F*). In the PL5, within-cRipple firing significantly decreased in NREM-preferring neurons, and similar trends were observed in REM-preferring neurons (Fig. 2*F*). In contrast, in the BLA, within-cRipple firing of NREM-preferring neurons significantly decreased but that of REM-preferring neurons significantly increased, with the difference between the two cell types approaching statistical significance (Fig. 2*F*). Regarding cortical spindles, REM-preferring neurons in the vCA1 and BLA significantly decreased their within-spindle firing, with the difference between NREM- and REM-preferring neurons in the vCA1 approaching statistical significance (Fig. 2*G*).

In summary, the FRs of both NREM- and REM-preferring neurons decreased during extended sleep in the vCA1, PL5, and BLA. In the vCA1 and BLA, NREM- and REM-preferring neurons showed differential changes in their firing activities during local fast network oscillations.

### REM- and NREM-preferring neurons were differentially modulated by fast network oscillations during sleep

The observed differences in FR changes within fast network oscillations between REM- and NREM-preferring neurons in the vCA1 and BLA prompted further investigation into how the sleep-state preference of individual neurons relates to their modulation by these oscillatory events. To quantify this relationship, FR gain during oscillatory events was calculated (Fig. 3*A*, Extended Data Fig. 3-1; see Materials and Methods). Across all brain regions examined, FR gain during SWRs was consistently higher in REM-preferring neurons than in NREM-preferring neurons (Fig. 3*B*, top). Moreover, a positive correlation was observed between the REM-preference index and FR gain during SWRs, confirming that this effect reflected the neurons’ NREM/REM preference rather than an artifact of neuronal classification (Fig. 3*B*, bottom). Notably, neurons that did not meet criteria for significant REM or NREM preference (“non-significant neurons”) were also included in the correlation analyses. These results indicate that neurons preferentially active during REM sleep exhibit greater FR gains during SWRs in sleep.

10.1523/ENEURO.0575-24.2025.f3-1 Figure 3-1 **Additional examples of firing rates aligned to fast network oscillation events** Firing rates (FRs) of example neurons aligned to power peaks of fast network oscillations (SWRs, HFOs, cRipples, and spindles). Similar to Fig. 3A, but these examples show the modulation of neuronal firing by oscillations detected in a *different* region than where the neuron was recorded. Peri-event FRs aligned with the peaks of individual oscillatory events are color-coded and sorted by FR peak. The red line represents the mean FR across oscillation events. The FR gain within the relevant oscillation is shown above each panel. Download Figure 3-1, TIF file.

10.1523/ENEURO.0575-24.2025.f3-2 Figure 3-2 **Statistical details for Fig. 3** N and R denote NREM- and REM-preferring neurons, respectively. n.a., not applicable. Download Figure 3-2, XLS file.

To further examine how other fast network oscillations modulate REM- and NREM-preferring neurons, the same analysis was applied to HFOs, cRipples, and cortical spindles. In the PL5 and BLA, FR gain during HFOs was significantly higher in REM-preferring neurons than in NREM-preferring neurons, whereas no such difference was observed in the vCA1 (Fig. 3*C*, top). Consistently, the REM-preference index correlated positively with FR gain during HFOs in the PL5 and BLA but not in the vCA1 (Fig. 3*C*, bottom). In contrast, FR gain during cRipples was higher in NREM-preferring neurons than in REM-preferring neurons in both the vCA1 and BLA, with a statistically significant difference in the vCA1 (Fig. 3*D*, top). Moreover, the REM-preference index negatively correlated with FR gain during cRipples in the vCA1 and BLA (Fig. 3*D*, bottom). Similar to SWRs and HFOs, FR gain during cortical spindles was significantly greater in REM-preferring neurons than in NREM-preferring neurons (Fig. 3*E*, top). Additionally, the REM-preference index positively correlated with FR gain during cortical spindles across all regions examined (Fig. 3*E*, bottom). Overall, these results suggest that SWRs, HFOs, and cortical spindles preferentially recruit REM-preferring neurons, whereas cRipples predominantly activate NREM-preferring neurons.

### Neurons with similar REM/NREM preferences exhibited greater within-region coactivity during fast network oscillations in sleep than those with differing preferences

The differential modulation of REM- and NREM-preferring neurons by fast network oscillations suggests the existence of distinct local networks that preferentially engage neurons with similar sleep-state preferences. Based on this, we hypothesized that neurons sharing the same sleep-state preference would coactivate more frequently during fast oscillatory events than neuron pairs with opposing preferences. To test this, neuronal coactivity was assessed using the coactivity *Z-*score (Cheng and Frank, 2008), which quantifies deviations from the expected frequency of coactivation under the assumption of independent activation (Fig. 4*A*, Extended Data Fig. 4-1; see Materials and Methods). A coactivity *Z-*score of 0 indicates chance-level coactivation, whereas positive and negative values indicate greater or lesser coactivation than expected, respectively. Neuron pairs were categorized into three groups based on their sleep preference: NREM-preferring pairs (NN), REM-preferring pairs (RR), and mixed pairs (NR) consisting of one NREM- and one REM-preferring neuron. During SWRs, both NN and RR pairs displayed significantly higher coactivity *Z*-scores than NR pairs in the PL5 (Fig. 4*B*, top middle), and RR pairs showed stronger coactivation than NR pairs in the BLA (Fig. 4*B*, top right). No significant differences among pair types were observed in the vCA1 (Fig. 4*B*, top left). To further examine the relationship between coactivation and sleep-state preference, the correlation between the coactivity *Z*-scores and the product of the REM-preference indices of each neuron pair was calculated. This analysis included pairs containing non-significant neurons. Since the product of REM-preference indices is positive for NN and RR pairs and negative for NR pairs, a positive correlation indicates greater coactivation among neurons with similar preferences. Indeed, significant positive correlations were found across all regions examined (Fig. 4*B*, bottom). These results suggest that, particularly in the PL5 and BLA, neurons with similar REM/NREM preferences exhibit stronger coactivation during SWRs compared with neurons with opposing preferences.

10.1523/ENEURO.0575-24.2025.f4-1 Figure 4-1 **Additional examples of coactivation of neuron pairs during fast network oscillations** Number of events in which example neuron pairs were coactivated, similar to Fig. 4A. Here, coactivation is shown during oscillations detected in a region *different* from where the neuron pair was recorded. The black curves show the theoretical distributions of the number of coactivation events, assuming that the neurons activate independently during oscillatory events. The observed number of coactivation events is indicated by the red vertical line, and the corresponding coactivity Z-score is displayed next to the line. Download Figure 4-1, TIF file.

10.1523/ENEURO.0575-24.2025.f4-2 Figure 4-2 **Statistical details for Fig. 4** NN: pairs of NREM-preferring neurons; RR: pairs of REM-preferring neurons; NR: pairs consisting of one NREM- and one REM-preferring neuron. n.a., not applicable. Download Figure 4-2, XLS file.

The same coactivity analysis was extended to HFOs, cRipples, and spindles. During HFOs, both NN and RR neuron pairs demonstrated significantly greater coactivity than NR pairs in the PL5, whereas in the BLA, RR pairs exhibited stronger coactivation than NR pairs (Fig. 4*C*, top). The coactivity *Z-*scores for HFOs positively correlated with the products of REM-preference indices across all regions examined (Fig. 4*C*, bottom). For cRipples, NN and RR pairs exhibited greater coactivity than NR pairs in the PL5, and RR pairs showed stronger coactivation than NR pairs in the BLA (Fig. 4*D*, top). Correspondingly, coactivity *Z*-scores for cRipples were positively correlated with REM-preference index products in both the PL5 and BLA (Fig. 4*D*, bottom). During cortical spindles, RR pairs exhibited stronger coactivation than NR pairs in the vCA1, while NN and RR pairs showed higher coactivity than NR pairs in the PL5 and BLA (Fig. 4*E*, top). The coactivity *Z*-scores for spindles showed significant positive correlations with REM-preference index products across all regions (Fig. 4*E*, bottom). Collectively, these findings suggest that neurons with similar REM or NREM preferences exhibit stronger within-region coactivity during fast network oscillations—including SWRs, HFOs, cRipples, and cortical spindles—in sleep than neurons with differing preferences.

### Neurons with similar REM/NREM preferences exhibited stronger cross-regional coactivity during fast network oscillations in sleep than those with differing preferences

Given the higher local coactivation in NN and RR pairs than in NR pairs during these oscillations, we hypothesized that cross-regional coactivation would similarly be stronger for neurons of the same preference type. To evaluate this, interregional coactivity *Z*-scores were calculated for neuron pairs categorized as NN (both NREM-preferring), RR (both REM-preferring), and NR/RN (mixed REM- and NREM-preferring, with the ordering of “*N*” and “*R*” denoting their respective regions) pairs. During SWRs, NN pairs showed stronger coactivation than NR and RN pairs in vCA1–PL5 (Fig. 5*A*, top left). In vCA1–BLA, NN pairs were more strongly coactivated than NR pairs, and RR pairs more than NR and RN pairs (Fig. 5*A*, top middle). Similarly, in PL5–BLA, both NN and RR pairs showed stronger coactivation than NR and RN pairs (Fig. 5*A*, top right). Additionally, coactivity *Z-*scores for SWRs correlated positively with REM-preference index products in vCA1–BLA and PL5–BLA cell pairs (Fig. 5*A*, bottom). Note that the product of REM-preference indices is positive for NN and RR pairs and negative for NR and RN pairs.

10.1523/ENEURO.0575-24.2025.f5-1 Figure 5-1 **Statistical details for Fig. 5** NN: NREM-preferring neuron pairs; RR: REM-preferring neuron pairs; NR and RN: pairs consisting of one NREM- and one REM-preferring neuron, where the order of ‘N’ and ‘R’ corresponds to the order of brain regions for the constituent neurons. n.a., not applicable. Download Figure 5-1, XLS file.

To determine whether NN and RR neuron pairs exhibit stronger cross-regional coactivation than NR and RN pairs during other types of fast network oscillations, the same analyses were extended to HFOs, cRipples, and spindles. During HFOs, NN and RR pairs showed stronger coactivation than RN pairs in vCA1–PL5 (Fig. 5*B*, top left). In vCA1–BLA, NN and RR pairs were more coactive than NR pairs, and RR pairs also exhibited greater coactivation than RN pairs (Fig. 5*B*, top middle). In PL5–BLA, NN pairs displayed stronger coactivation than NR pairs, while RR pairs were more coactive than both NR and RN pairs (Fig. 5*B*, top right). Coactivity *Z*-scores for HFOs were positively correlated with the products of the REM-preference indices across all three region pairs: vCA1–PL5, vCA1–BLA, and PL5–BLA (Fig. 5*B*, bottom). During cRipples, no significant differences in coactivity were observed among vCA1–PL5 neuron pair types (Fig. 5*C*, top left). However, in vCA1–BLA, RR pairs showed stronger coactivation than both NR and RN pairs (Fig. 5*C*, top middle). In PL5–BLA, both NN and RR pairs exhibited greater coactivation than NR and RN pairs (Fig. 5*C*, top right). Coactivity *Z-*scores for cRipples showed significant positive correlations with REM-preference index products across all three region pairs (Fig. 5*C*, bottom). During spindles, RR pairs were more coactive than RN pairs in vCA1–PL5, and more so than both NR and RN pairs in vCA1–BLA (Fig. 5*D*, top left and middle). In PL5–BLA, NN and RR pairs demonstrated stronger coactivation than NR and RN pairs (Fig. 5*D*, top right). Coactivity *Z*-scores for spindles also positively correlated with REM-preference index products in all region pairs examined (Fig. 5*D*, bottom). Overall, these results indicate that cross-regional coactivations were stronger in NN and RR pairs than in NR and RN pairs during various fast network oscillations in sleep.

Based on the observation that cross-regional pairs consisting of the same cell types showed stronger coactivity than those consisting of both cell types (Fig. 5*A–D*), we hypothesized that the proportion of NREM-preferring neurons active in one region during an oscillatory event would positively correlate with the FRs of NREM-preferring neurons and negatively with the FRs of REM-preferring neurons in another region during the same event. To test this, NREM-preferring neuron response indices were calculated for SWRs, HFOs, cRipples, and spindles. These indices represent the Pearson's correlation coefficients between the proportion of NREM-preferring neurons among neurons that are active during individual oscillatory events of interest in the region where these events were detected and the FRs of individual neurons in another region during the same events (Harvey et al., 2023; see Materials and Methods). Consistent with the hypothesis, NREM-preferring neuron response indices for SWRs were higher in NREM-preferring neurons than in REM-preferring neurons in the PL5 and BLA (Fig. 5*E*), and the indices for HFOs were higher in NREM-preferring neurons than in REM-preferring neurons in the vCA1 and PL5 (Fig. 5*F*). The indices for cRipples were higher for NREM-preferring neurons in the BLA (Fig. 5*G*). The indices for spindles were higher for NREM-preferring neurons in the vCA1 and BLA (Fig. 5*H*). These findings suggest that a higher proportion of NREM- or REM-preferring neurons among active neurons in one region correlates with higher FRs of the same neuron type in another region during sleep-related oscillatory events.

### REM- versus NREM-preferring neurons were differentially modulated by awake SWRs and shock stimuli during wakefulness

Previous findings demonstrated differential responses of REM- and NREM-preferring neurons to fast network oscillations during sleep. Since such oscillations are implicated in memory consolidation by reactivating activity patterns from wakefulness, we hypothesized that these neuron types would also exhibit distinct firing patterns during wakefulness. To evaluate this, mean FRs during wakefulness, encompassing all awake epochs across behavioral and hc sessions, were calculated. Given that both REM sleep and active wakefulness share desynchronized forebrain activity (Llinás and Paré, 1991) and hippocampal theta oscillations (Buzsáki, 2002), it was expected REM-preferring neurons would be more active than NREM-preferring neurons during wakefulness. Contrary to this expectation, NREM-preferring neurons exhibited higher FRs than REM-preferring neurons during wakefulness across all examined regions (Fig. 6*A*). Furthermore, FRs during wakefulness showed a significant negative correlation with the REM-preference indices in all regions examined (Fig. 6*B*). These findings indicate that NREM-preferring neurons display higher overall activity than REM-preferring neurons during wakefulness.

10.1523/ENEURO.0575-24.2025.f6-1 Figure 6-1 **Statistical details for Fig. 6** N and R indicate NREM- and REM-preferring neurons, respectively. n.a., not applicable. Download Figure 6-1, XLS file.

Considering that SWRs during sleep modulate REM- and NREM-preferring neurons differently (Fig. 3*B*), their modulation by SWRs during wakefulness, which included all awake epochs in behavioral and hc sessions, was examined (Fig. 6*C*). REM-preferring neurons showed a greater increase in firing than NREM-preferring neurons during awake SWRs in all regions examined (Fig. 6*C*, top), similar to the pattern observed during sleep (Fig. 3*B*). Furthermore, REM-preference indices positively correlated with FR gains induced by awake SWRs (Fig. 6*C*, bottom). These results suggest that REM-preferring neurons are more strongly modulated by awake SWRs than NREM-preferring neurons, consistent with their modulation by sleep SWRs.

Given the involvement of awake SWRs in memory acquisition and cognitive functions (Foster and Wilson, 2006; Jadhav et al., 2012), and of sleep SWRs in memory consolidation (Girardeau et al., 2009; Buzsáki, 2015), the observed greater FR gain of REM-preferring neurons during SWRs in both wakefulness (Fig. 6*C*) and sleep (Fig. 3*B*) led to hypothesis that REM-preferring neurons may more robustly encode behaviorally salient events. To test this, firing responses to shock stimuli during cued fear conditioning sessions were analyzed. Although both REM- and NREM-preferring neurons exhibited increased firing during shock compared with baseline (before the first CS), REM-preferring neurons displayed significantly greater increases in the vCA1 and BLA (Fig. 6*D–F*). Furthermore, FR gain in response to shock stimuli positively correlated with REM-preference index in PL5 and BLA (Fig. 6*F*, bottom). These results indicate that during shock presentation, the REM-preferring neurons were more strongly activated than the NREM-preferring neurons.

In summary, REM-preferring neurons were more strongly modulated by both SWRs and shock stimuli during wakefulness than NREM-preferring neurons, indicating potential differences in their roles in memory acquisition.

### Neurons with differing REM/NREM preferences exhibited stronger cross-regional coactivity than neurons with similar preferences during awake SWRs in conditioning session

Although neurons preferring REM and NREM sleep differed in response magnitude, both types responded to salient stimuli during wakefulness (Fig. 6*D–F*), suggesting potential functional interactions between them. Previous analyses revealed distinct within- and cross-regional coactivation patterns between REM- and NREM-preferring neurons during sleep (Figs. 4, 5). Based on these findings, we hypothesized that REM- and NREM-preferring neurons would also exhibit transient within- and cross-regional coactivation during wakefulness in response to salient experiences. To investigate this hypothesis, within- and cross-regional coactivity was compared during awake SWRs recorded in baseline and conditioning sessions. Because neuronal reactivation during sleep is most prominent immediately following an experience (Kudrimoti et al., 1999), for this analysis, REM- and NREM-preferring neurons were classified independently for the baseline and conditioning sessions, based on their firing preference in the hc session immediately following each behavioral session.

First, within-regional coactivity during the baseline and conditioning sessions was examined (Extended Data Figs. 7-1, 7-2). During baseline sessions, within-regional coactivity of RR neuron pairs was significantly stronger than that of NR pairs in PL5 (Extended Data Fig. 7-1*A*). Additionally, coactivity *Z*-scores for awake SWRs positively correlated with the products of REM-preference indices within both PL5 and BLA (Extended Data Fig. 7-1*A*). During conditioning sessions, within-regional coactivity of NN pairs was significantly stronger than that of NR pairs in vCA1, while RR pairs again exhibited stronger coactivity than NR pairs in PL5 (Extended Data Fig. 7-1*B*). Coactivity *Z*-scores for awake SWRs remained positively correlated with the REM-preference index products in PL5 (Extended Data Fig. 7-1*B*). These results suggest that within-regional coactivation of neurons with similar sleep-state preferences was either comparable with or stronger than that of neurons with differing preferences during both baseline and conditioning sessions.

Next, cross-regional coactivity was assessed (Fig. 7). During baseline sessions, no significant differences in coactivity were observed among neuron pair types in vCA1–PL5 and vCA1–BLA (Fig. 7*A*, top left and middle). However, in PL5–BLA, RN pairs displayed significantly weaker coactivity than both NN and RR pairs (Fig. 7*A*, top right). Similarly, coactivity *Z*-scores for awake SWRs positively correlated with the products of REM-preference indices in PL5–BLA pairs (Fig. 7*A*, bottom). These results suggest that cross-regional coactivation of neurons with similar sleep-state preferences was either comparable with or stronger than that of neurons with opposing preferences during baseline sessions.

10.1523/ENEURO.0575-24.2025.f7-1 Figure 7-1 **Within-region coactivity of excitatory neuron pairs during awake SWRs** Within-region coactivity *Z*-scores for awake SWRs are shown separately for the baseline (A) and conditioning (B) sessions in vCA1, PL5, and BLA. Top panels: Mean coactivity *Z*-scores for each cell pair type. The NN, RR, and NR pairs are shown separately. Error bars represent standard errors of the mean (SEM). Bottom panels: Relationships between the products of REM-preference indices and coactivity *Z*-scores. Each dot represents a cell pair. Linear regression lines are shown in black, and the Spearman’s rank-order correlation coefficients are shown in the upper right. *p < 0.05, **p *<* 0.01, ***p *<* 0.001; Top panels: Post hoc Steel–Dwass test following the Kruskal–Wallis test. Bottom panels: Significance of Spearman’s rank-order correlation. Neuron pairs including non-significant neurons were also included in the correlation analysis. See Extended Data Fig. 7-2 for the number of cell pairs analyzed and detailed statistical results. *Abbreviations*: NN: NREM-preferring neuron pairs; RR: REM-preferring neuron pairs; NR: Pairs of NREM- and REM-preferring neurons. Download Figure 7-1, TIF file.

10.1523/ENEURO.0575-24.2025.f7-2 Figure 7-2 **Statistical details for Extended Data Fig. 7-1** NN, NREM-preferring neuron pairs; RR, REM-preferring neuron pairs; NR: pairs consisting of one NREM- and one REM-preferring neuron. n.a., not applicable. Download Figure 7-2, XLS file.

10.1523/ENEURO.0575-24.2025.f7-3 Figure 7-3 **Statistical details for Fig. 7** NN: pairs of NREM-preferring neurons; RR: pairs of REM-preferring neurons; NR and RN: pairs consisting of one NREM- and one REM-preferring neuron, where the order of ‘N’ and ‘R’ corresponds to the order of brain regions for the constituent neurons. n.a., not applicable. Download Figure 7-3, XLS file.

In contrast, during conditioning sessions, cross-regional coactivation was stronger in NR and RN pairs than that in NN and RR pairs in several region pairs. In vCA1–PL5, NR pairs exhibited significantly stronger coactivity than RR pairs (Fig. 7*B*, top left). In vCA1–BLA, both NR and RN pairs showed significantly stronger coactivity than RR pairs (Fig. 7*B*, top middle). In PL5–BLA, RN pairs exhibited stronger coactivity than RR pairs (Fig. 7*B*, top right). Additionally, coactivity *Z*-scores for awake SWRs negatively correlated with the products of REM-preference indices in vCA1–PL5, vCA1–BLA, and PL5–BLA pairs (Fig. 7*B*, bottom).

These results suggest that cross-regional coactivation, but not within-regional coactivation, during awake SWRs in conditioning sessions was transiently enhanced in neuron pairs with differing REM/NREM preferences. In contrast, within-regional coactivity remained stronger in neuron pairs with similar preferences. These findings suggest that REM- and NREM-preferring neurons engage in dynamic inter-regional interactions during salient experiences in wakefulness, potentially supporting memory formation.

## Discussion

This study demonstrates that neuronal firing modulation by sleep states is highly heterogeneous, with local circuits comprising distinct populations of REM- and NREM-preferring neurons (Fig. 1). While both types exhibited reduced firing during sleep, their activity within fast network oscillations markedly differed (Fig. 2). Moreover, firing modulation by sleep states correlated with modulation by fast network oscillations (Figs. 3, 8*A*), suggesting that REM- and NREM-preferring neurons are differentially regulated by fast oscillations but are under similar homeostatic control during sleep. Furthermore, neurons with similar REM/NREM preferences showed stronger within- and cross-regional coactivation during fast oscillations in sleep than neurons with differing preferences (Figs. 4, 5, 8*B*,*C*). In contrast, during awake SWRs in conditioning sessions, cross-regional coactivation was more pronounced between neurons with differing REM/NREM preferences (Figs. 7*B*, 8*C*). These findings suggest that interactions between REM- and NREM-preferring neurons are restricted during sleep but become transiently enhanced during initial memory encoding, such as during fear conditioning.

SWRs can induce both LTD (Norimoto et al., 2018) and LTP (Buzsáki et al., 1987; King et al., 1999), leading to an overall reduction in neuronal firing (Miyawaki and Diba, 2016) and facilitating memory consolidation (Barnes and McNaughton, 1985; Girardeau et al., 2009). In addition, other fast network oscillations, such as HFOs, cRipples, and spindles, have been implicated in synaptic plasticity (Maingret et al., 2016; Khodagholy et al., 2017; Miyawaki and Mizuseki, 2022). Since these network oscillations modulate REM- and NREM-preferring neurons differently (Fig. 3), they may drive distinct synaptic plasticity mechanisms depending on REM/NREM preference. This differential modulation could support both the homeostatic downregulation of neuronal activity and memory consolidation during sleep.

The classification of REM- and NREM-preferring neurons remained consistent for at least a day (Fig. 1*G*,*H*, Extended Data Figs. 1-4, 1-6), suggesting inherent differences in their intrinsic properties. Differences in responsiveness to neuromodulators such as serotonin, noradrenaline, and acetylcholine may underlie the variation in NREM/REM preference. Serotonergic, noradrenergic, and cholinergic neurons project from the brainstem to regions such as the hippocampus, prefrontal cortex, and amygdala (Azmitia et al., 1978; Berger et al., 1979; Woolf et al., 1983, 1984; Jacobs and Azmitia, 1992; Uematsu et al., 2015), with activity levels varying across sleep state (Aston-Jones and Bloom, 1981; Jacobs and Azmitia, 1992; Hobson and Pace-Schott, 2002). A specific serotonin receptor subtype has been implicated in fear memory retrieval (Ohmura et al., 2016), whereas certain adrenergic and acetylcholine receptors are involved in fear memory formation (Lazzaro et al., 2010; Mineur et al., 2020). These findings imply that neuron-specific receptor expression patterns shape neuronal responses during fear memory processing. Consistent with this possibility, REM-preferring neurons in the vCA1 and BLA exhibited significantly higher FR gain during shock presentation than NREM-preferring neurons (Fig. 6*F*), suggesting distinct neuromodulator receptor expression patterns in NREM- and REM-preferring neurons.

Another potential factor influencing REM/NREM preference is inhibitory input from local networks. In the cortex, parvalbumin-positive (PV+) interneurons show higher activity during REM sleep, whereas somatostatin-positive (SOM+) interneurons more active during NREM sleep (Niethard et al., 2016). Furthermore, PV+ interneurons preferentially inhibit specific layer 5 pyramidal neurons, whereas SOM+ interneurons target a broader neuronal population (A. T. Lee et al., 2014). Thus, NREM-preferring neurons in PL5 may be more strongly inhibited by PV+ interneurons than REM-preferring neurons. In the hippocampus, PV+ interneurons inhibit pyramidal neurons in a nonuniform manner (S. H. Lee et al., 2014) and a similar diversity in inhibitory control may exist in vCA1 and BLA, contributing to the observed differences in sleep-state preference among individual neurons.

FR modulations by sleep states and those by fast network oscillations were diverse and correlated (Figs. 3, 8*A*). Furthermore, high- and low-FR neurons are differently modulated by sleep-state changes (Watson et al., 2016; Miyawaki et al., 2019), which may be related to variability in synaptic strength changes. Thus, the diversity in FR modulation across sleep states may reflect variations in synaptic strength adjustments driven by network oscillations—an idea that warrants further investigation.

REM-preferring neurons are characterized by low FRs during wakefulness and substantial firing gains during SWRs in both sleep and wakefulness (Figs. 3*B*, 6*A–C*, 8*A*). Notably, low-firing neurons with high SWR gains are known to exhibit strong learning-related plasticity (Grosmark and Buzsáki, 2016). In addition, the high cholinergic activity during REM sleep facilitates LTP induction (Hasselmo and Bower, 1993), suggesting that spike-dependent LTP processes may be prominent at the synapses of REM-preferring neurons than those of NREM-preferring neurons. Given that elevated cholinergic tone during REM sleep is crucial for memory consolidation (Rasch et al., 2009), enhanced LTP induction at REM-preferring neuron synapses likely contributes to this process. In addition, REM-preferring neurons exhibited stronger activation in response to shock stimuli than NREM-preferring neurons (Fig. 6*F*). These findings suggest that REM-preferring neurons are more plastic and play important roles in memory formation.

During fast network oscillations in sleep, both within- and cross-regional coactivations of neuron pairs with similar REM/NREM preferences were more robust than those with differing preferences (Figs. 4, 5, 8*B*,*C*). Given that population bursts during SWR promotes Hebbian plasticity (Buzsáki, 1989), this pattern suggests that coactivation during fast network oscillations in sleep may facilitate the strengthening of connections between neurons—both within and across regions—that share REM/NREM preference. In contrast, during awake SWRs in conditioning sessions, cross-regional coactivation was more pronounced between neurons with differing REM/NREM preferences than between neurons with similar preferences (Figs. 7*B*, 8*C*). Given that hippocampal–cortical reactivation is most prominent during awake SWRs during initial learning in novel environments (Tang et al., 2017), this transient cross-regional coactivation of neurons with differing REM/NREM preferences may play an important role in the early stages of memory encoding. In the current dataset, behavioral responses during conditioning and cue-retention sessions were similar across animals, likely due to the relatively strong shock stimuli employed. These stereotyped behavioral patterns limited our ability to assess potential correlations between cross-regional coactivation and fear learning. Future studies should explore how inter-regional coactivation dynamics relate to memory acquisition under milder shock conditions.

In conclusion, although both NREM- and REM-preferring neurons exhibited decreased firing during sleep, they responded differentially to fast network oscillations and shock stimuli. These differences may support distinct forms of synaptic plasticity, shaping both local and global networks organized by REM/NREM preference. Such mechanisms likely contribute to the dual roles of sleep in maintaining homeostatic regulation of neuronal excitability and promoting experience-dependent synaptic modifications. However, the direct link between FR changes and synaptic plasticity remains to be elucidated. Moreover, NREM- and REM-preferring neurons may encode distinct types of information during wakefulness, a possibility that warrants systematic investigation. The extent to which learning and memory processes modulate REM/NREM preference at the single-neuron level, as well as the dynamics of within- and cross-regional coactivation among these neuronal populations, remains unclear. Future studies addressing these questions will help clarify the distinct contributions of sleep states to both homeostatic control and memory consolidation.

## Synthesis

Reviewing Editor: Nathalie Ginovart, University of Geneva Decisions are customarily a result of the Reviewing Editor and the peer reviewers coming together and discussing their recommendations until a consensus is reached. When revisions are invited, a fact-based synthesis statement explaining their decision and outlining what is needed to prepare a revision will be listed below. The following reviewer(s) agreed to reveal their identity: Andres Grosmark.

The reviewers expressed interest in your manuscript and agreed that it provides new insights into neuronal recruitment during REM and NREM sleep across three brain regions. However, a few points were raised that should be addressed in a revision to improve the clarity of the manuscript and potentially enhance its impact. In addition to providing missing details, additional analyses should be performed to assess regional differences in preference stability while accounting for cell number, and to examine learning-related changes in preference stability.

Please find the reviewer's comments below:

Reviewer 1:

While mammalian sleep stages can be classified into two major stages, namely NREM and REM sleep, it remains unclear how neural ensembles in different brain regions behave across sleep stages. To address this important issue, the authors built on their previous publication (Miyawaki and Mizuseki, 2022) and analyzed the same dataset.

By classifying single units into two functional classes ("REM/NREM-preferring neurons") in three brain regions (vCA1, PL5 and BLA) of rats, the authors comprehensively described their functional properties, such as firing rate changes, modulation associated with four fast oscillations (SWR, HFO, cortical ripples, sleep spindles), intra- and inter-regional coactivity, and activity during wakefulness.

Their main observations are

1. Homeostatic firing rate changes in both neuron types during sleep

2. Variable modulations of their activity across four fast oscillations and brain regions

3. In each brain region, co-activity during fast oscillations is generally higher in pairs of the same functional classes than in pairs of different classes across brain regions.

4. The same applies to inter-regional co-activity

5. During wakefulness, REM-preferring neurons are more excitable

6. Inter-regional co-activity during wakefulness exhibits differential patterns between the baseline and conditioning sessions.

The results are novel and supported by the large sample size and the authors did an excellent job in their data analysis in general. Since this is a descriptive study, the presentation could be improved to attract a larger audience.

I believe that addressing the following issues will refine the manuscript.

MAJOR

General comments

It is better to make this manuscript stand alone. This means that the authors should make extra effort to craft the manuscript so that readers do not need to read their previous paper.

Additionally, providing extra explanatory figures or diagrams will help readers understand each key metric, such as the REM-preference index, Gain and Coactivity.

Introduction

In the current version, it is not easy to understand the rationale of this study. The authors may reconsider the flow based on their main results.

For example, after providing a general introduction, the authors may want to (1) summarize what we know about neural firing between NREM and REM sleep, (2) briefly describe four fast oscillations and their relation to sleep-wake cycles, and (3) summarize and critically analyze the previous study (Miyawaki and Mizuseki, 2022) to identify the knowledge gap.

At least please make sure that the authors justify their analyses more clearly by setting up a research context properly.

Methods

The following points need to be clarified:

1. 17 hours recording - give the time range in Zeitgeber time

2. What type of silicon probes was used?

3. Clarify the inclusion criteria for single units.

4. The definition of excitatory and inhibitory connections should be described in more detail for reproducibility. Were CCGs computed only within neurons in the same region? Providing an explanatory figure with an example plot is helpful.

5. For sleep scoring, what was the window size?

6. Various thresholds for event detection or cell classification seem arbitrary. Where possible, it would be helpful to justify them.

7. The stats of sleep states should be given as a table.

Results

At the beginning, please describe the following basic information: (1) the sample size (# of animals, # of analyzed single units per region), and (2) the frequency of the four fast oscillations across sessions and sleep stages.

In Figure 1, since the classification of REM/NREM-preferring neurons is fundamental for all the following analyses, providing a better explanatory figure is essential. In the current Fig. 1B, showing the time-normalized firing profiles is misleading. Readers might misunderstand that the index was calculated based on those profiles.

In Fig. 1B &C, the tick positions are confusing. When did state transitions happen?

Related to Fig. 1F, the authors may also show equivalent scatter plots for each brain region as a supplementary figure.

In Fig. 1G, was the correlation coefficient Pearson's?

In Fig. 2B and other figures, wherever non-significant results were obtained, please provide the exact p-value, rather than using a different symbol. To make the claim stronger, computing Cohen's d helps.

Before showing Figure 3, provide example plots to show what each detected fast oscillation looks like and how these two functional classes are modulated.

An analysis of intra-regional coactivity during awake SWRs may make this study more comprehensive.

Discussion

Provide a summary before discussing the findings. Providing a supplementary figure or table to summarize all the main findings would be helpful.

Additionally, discuss the limitations of the study. Since the authors did not look at detailed neural activity in individual hc sessions, it is difficult to tell exactly what changes in neural activity are associated with learning and memory.

MINOR

Line 1000. Is this intra-regional coactivity, rather than "inter-regional"?

Reviewer 2:

In their manuscript "Firing activities of REM- and NREM-preferring neurons are differently modulated by fast network oscillations and behavior in the hippocampus, prelimbic cortex, and amygdala" the authors present the results of an extensive and novel set of analyses of sleep related neural activity during sleep. The researchers conducted 17-hour continuous recordings of single-unit activity and local field potentials in three brain regions (ventral hippocampal CA1, prelimbic cortex layer 5, and basolateral amygdala) of fear-conditioned rats. They report that the REM vs. NREM firing rate preference of individual neurons, correlates with their recruitment to several brain oscillations, as well as to their inter- and intra- regional coactivity patterns. While the current manuscript is a reanalysis of a previously published dataset, the analysis presented in the manuscript is sophisticated and the results appear novel and interesting. We do have some concerns regarding the characterization of the REM vs. NREM preferring cells, as well as some minor concerns regarding the data presentation and discussion. However, overall the manuscript is in good shape and is close to ready for publication.

Major concern:

The entire manuscript relies on the characterization of cells as REM vs. NREM-preferring. The authors state that "[t]he classification of REM- and NREM-preferring neurons was consistent, at least within a day (Fig. 1F, G), suggesting that the intrinsic properties of these cells are different" (lines 659-60). However, Fig 1G shows that the stability of vCA1 REM/NREM preference uniquely decays within the 17 hour window such that hc0 and hc1 are not significantly correlated to hc3 and hc4. If it were found that the dynamics of vCA1 cells shifted on a more rapid timescale than those of BLA or PL5 this would represent important additional context for the current study. It would also present a significant further novel result, potentially linking hippocampal sleep activity to its theorized role in rapid (episodic) learning. However, it may be that the apparent rapid 'decay' of REM/NREM preference stability across hc epochs is instead due to the relatively low number of vCA1 as compared to PL5 or BLA cells. Therefore, additional analyses should be carried out to test whether the differences in preference stability observed across regions are significant, while controlling for differences in cell numbers across these regions. Related, to question of whether the preference is intrinsic or not, the authors should characterize whether the stability of the NREM/REM preference changes as a function of learning. For example, the authors could directly compare the stability of these preferences between the hc1 to hc2 (learning) versus hc0 to hc1 (no learning) epochs. In short: the rest of the manuscript relies on the REM/NREM preference dichotomy - so any further insight into the dynamics of this dichotomy would strengthen all of the author's subsequent conclusions, and potentially put them into an interesting new light, potentially emphasizing their relationship to learning and/or slow, putatively homeostatic, changes in neural dynamics.

Minor concerns:

The way the figures are laid out is very dense and difficult to parse. One major and relatively easy improvement would be the introduction of some white space between the groups of panels belonging to the same figure letter (this suggestion primarily applies to figures 4, 5 and 6). Likewise, wherever possible titles should be added to groups of related panels - this is already done in figure 5, but these should be made larger and added to figure 6.

While it may be an issue of style, the results are presented so directly and succinctly that they are quite hard to follow. While, major conclusions should be left for the Discussion section, in the Results section it is appropriate to 1) give a short summary of the rationale why a particular experiment or analysis was carried out, and 2) provide a brief interpretation of the results of said experiments or analyses. This isn't a requirement, but it would substantially increase the intelligibility and therefore impact of the current study.
