# Supplementary figures and images for: Firing Activities of REM- and NREM-Preferring Neurons Are Differently Modulated by Fast Network Oscillations and Behavior in the Hippocampus, Prelimbic Cortex, and Amygdala
Source: eNeuro. 2025 May 23;12(5):ENEURO.0575-24.2025. doi: 10.1523/ENEURO.0575-24.2025 (PMC12118951; doi:10.1523/ENEURO.0575-24.2025)

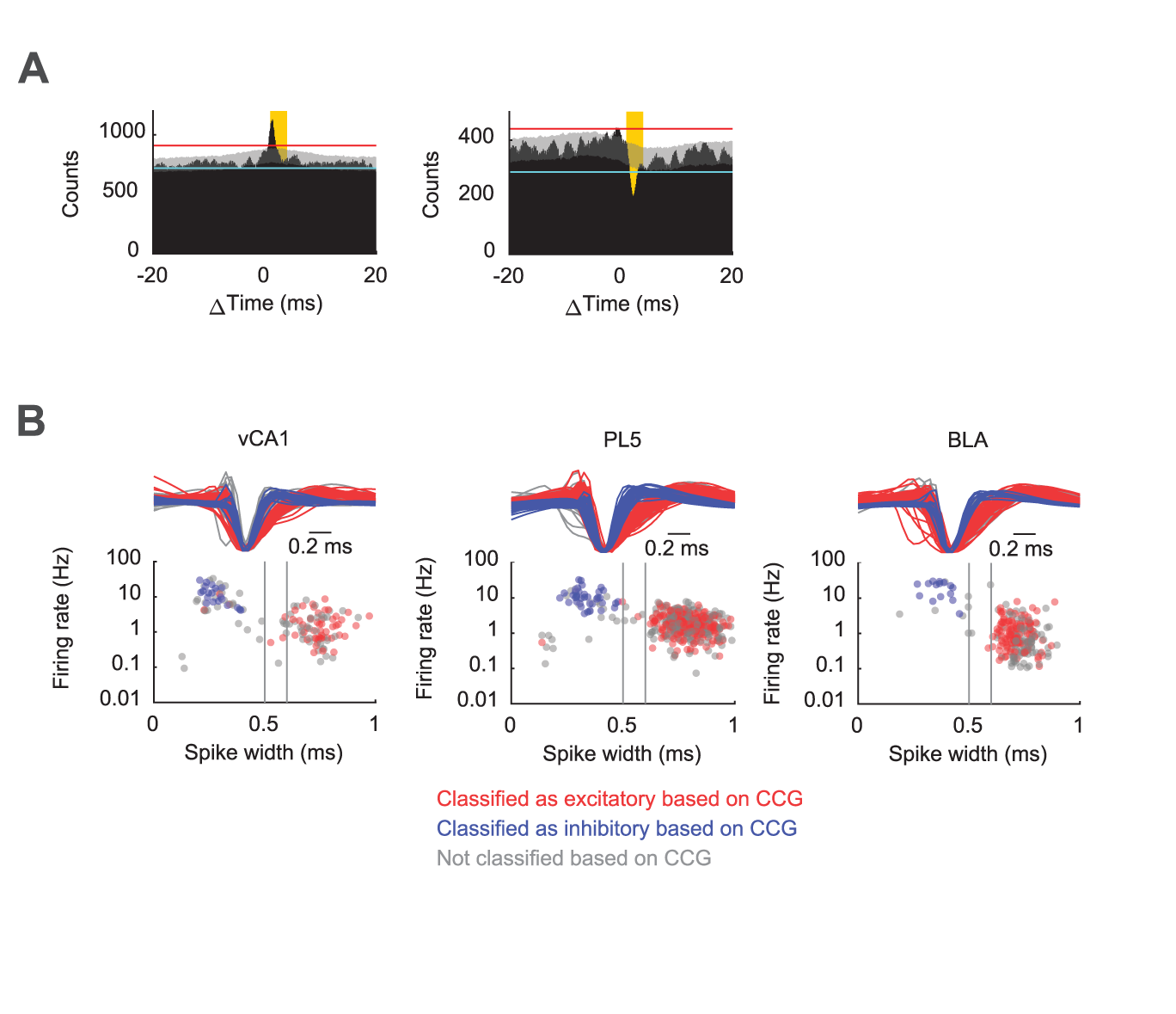

Supplement: Table 1-1 — Classification of excitatory and inhibitory neurons (A) Example cross-correlograms (CCGs) of spike times from neuron pairs showing significant spike transmission (left) and suppression (right). Red and cyan lines indicate the 99% confidence intervals (CIs) for detected CCG peaks and troughs within the [–5, + 5] ms range, respectively. The gray band represents the 99% CI at each time point, obtained by jittering spike timings. The orange background highlights the [+1, + 4] ms period used to evaluate the significance of peaks and troughs. (B) Mean waveforms of all recorded neurons in each brain region (top), and scatter plots of spike width versus mean firing rate (bottom). The vertical scales of the mean waveforms are normalized by spike amplitudes. Colors represent cell types: red for putative excitatory neurons, blue for putative inhibitory neurons, and gray for non-classified neurons. (A) and (B): Reproduced from Miyawaki and Mizuseki (2022) under a CC-BY 4.0 license. Download Table 1-1, TIF file. [file eneuro-12-ENEURO.0575-24.2025-s022.tif]

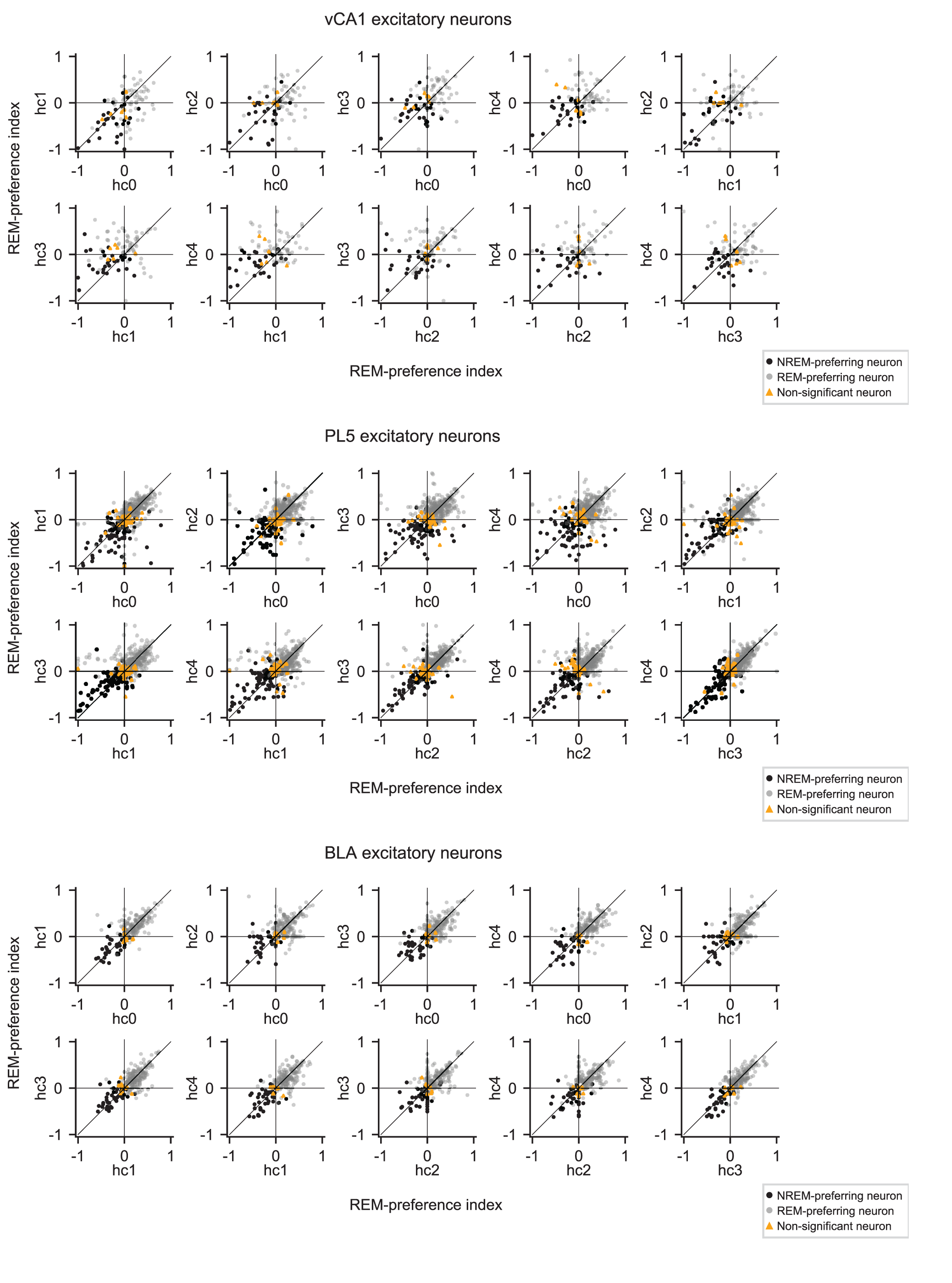

Supplement: Figure 1-4 — Comparison of REM-preference indices of excitatory neurons across home cage sessions Same as Fig. 1G, but excitatory neurons in the vCA1, PL5, and BLA are shown separately. REM-preference indices of excitatory neurons were calculated within each home cage (hc) session. REM/NREM preferences remained largely stable across sessions. Dot colors represent neuron types classified based on all concatenated sleep epochs, as in Fig. 1G: black circles indicate NREM-preferring neurons, gray circles indicate REM-preferring neurons, and orange triangles indicate non-significant neurons. Statistical details are provided in Extended Data Fig. 1-5. Download Figure 1-4, TIF file. [file eneuro-12-ENEURO.0575-24.2025-s005.tif]

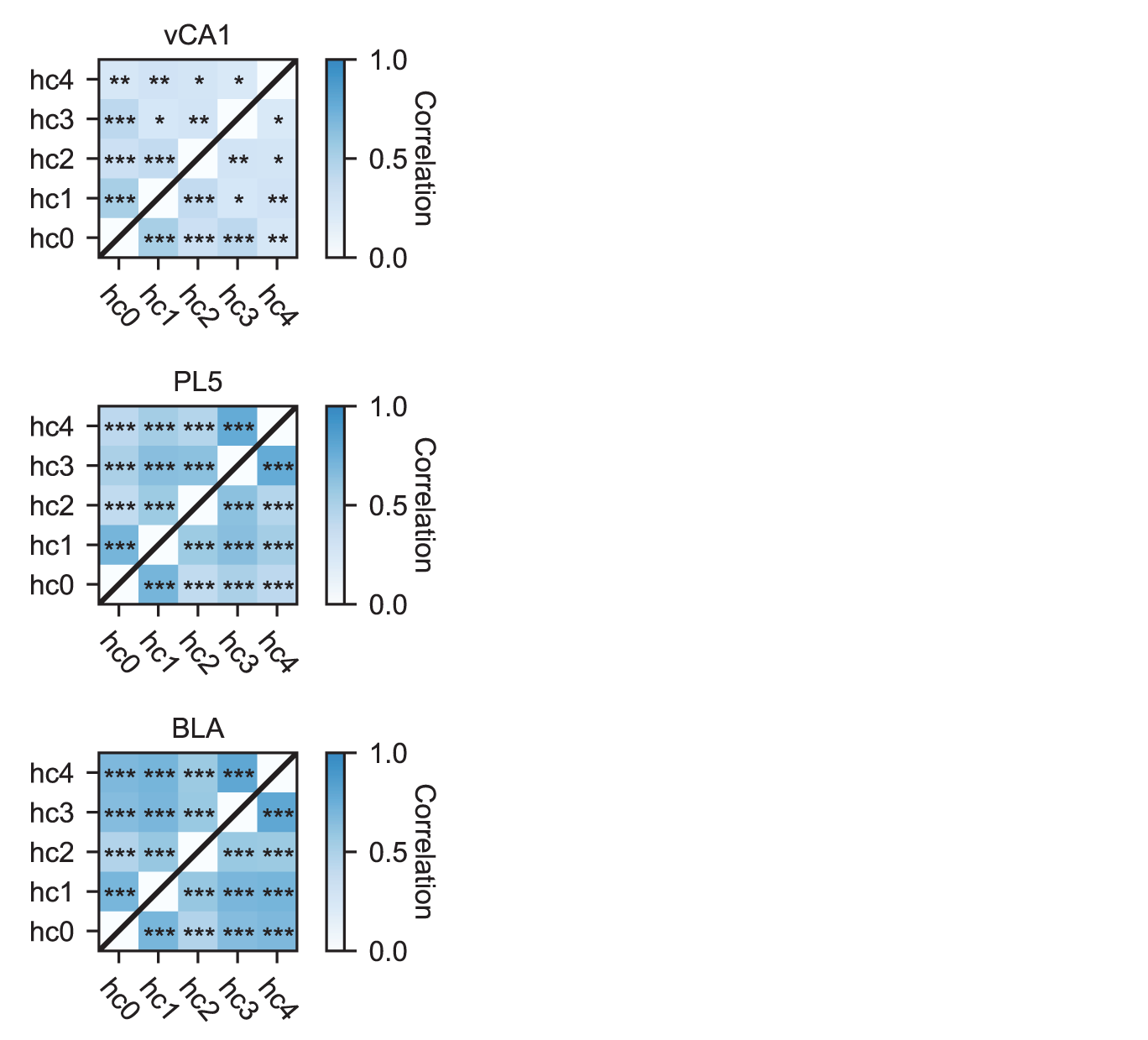

Supplement: Figure 1-6 — Correlation of REM-preference indices across home cage sessions using bootstrapping Same as Fig. 1H, but Spearman’s rank-order correlation coefficients of REM-preference indices between home cage sessions in the vCA1, PL5, and BLA were estimated using a bootstrapping method. An equal number of neurons (n = 92) was sampled in each brain region to control for differences in cell counts. Medians of the bootstrapped rank-order correlation coefficients are shown. *p < 0.05, **p < 0.01, ***p < 0.001. See Extended Data Fig.1-7 for statistical details. Download Figure 1-6, TIF file. [file eneuro-12-ENEURO.0575-24.2025-s007.tif]

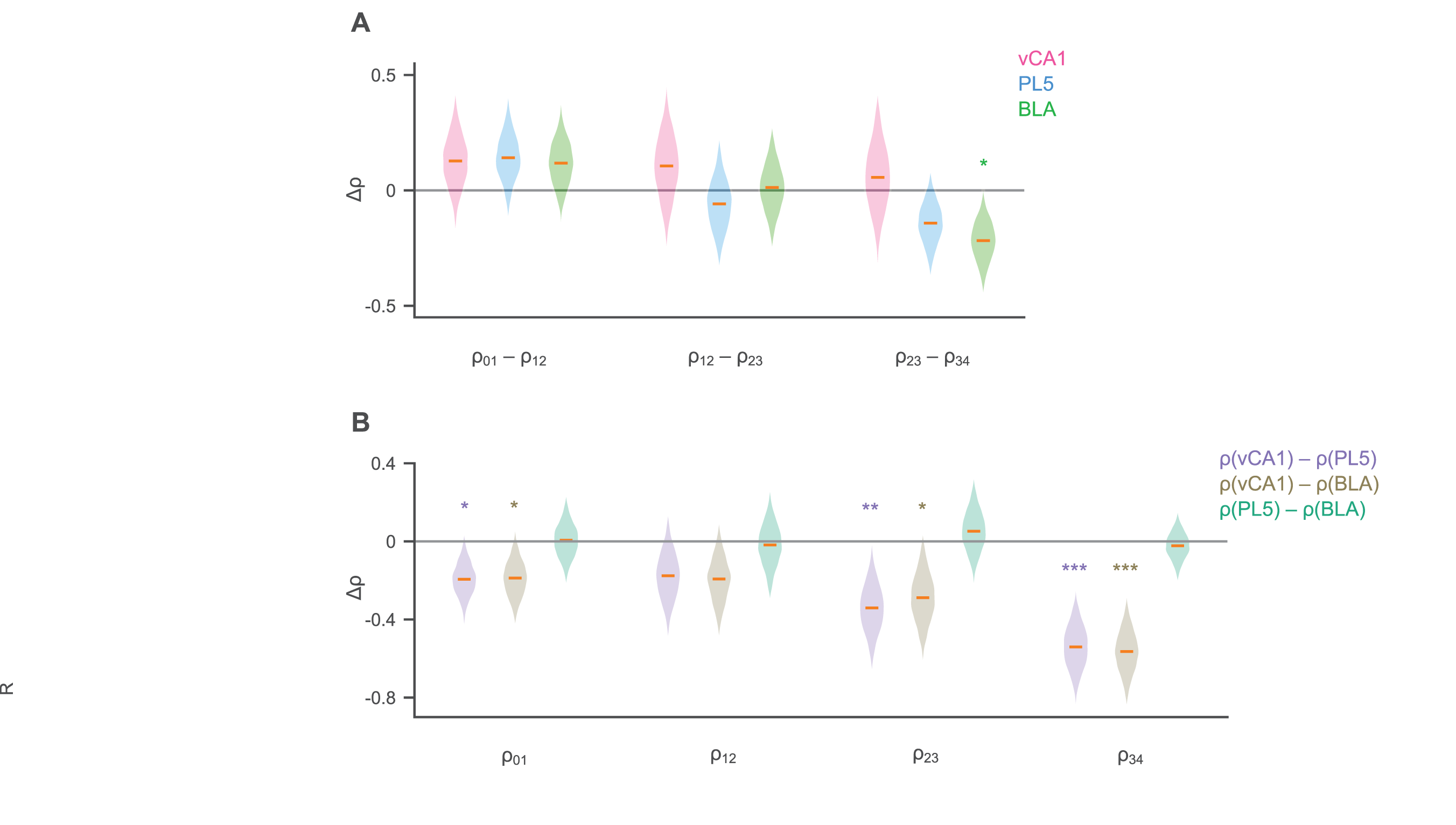

Supplement: Figure 1-8 — Comparison of Spearman’s rank-order correlation of REM-preference indices estimated via bootstrapping (A) For each brain region, an equal number of neurons (n = 92) were resampled with replacement, and Spearman’s rank-order correlation coefficients of REM-preference indices were calculated between temporally adjacent home cage (hc) sessions. The difference in correlation coefficients between hc session pairs was then computed. This procedure was repeated 5,000 and the resulting distributions with their medians (orange bars) are shown. *p < 0.05. See Extended Data Fig. 1-9 for statistical details. (B) For each pair of brain regions, 92 neurons were resampled with replacement per region. Spearman’s rank-order correlation coefficients of REM-preference indices between temporally adjacent home cage sessions were computed for each region, and inter-region differences were calculated. This procedure was also repeated 5,000 times and the resulting distributions with their medians (orange bars) are shown. *p < 0.05, **p < 0.01, ***p < 0.001. See Extended Data Fig. 1-9 for statistical details. Spearman’s rank-order correlation coefficient between hcX and hcY is denoted as ρXY. Download Figure 1-8, TIF file. [file eneuro-12-ENEURO.0575-24.2025-s009.tif]

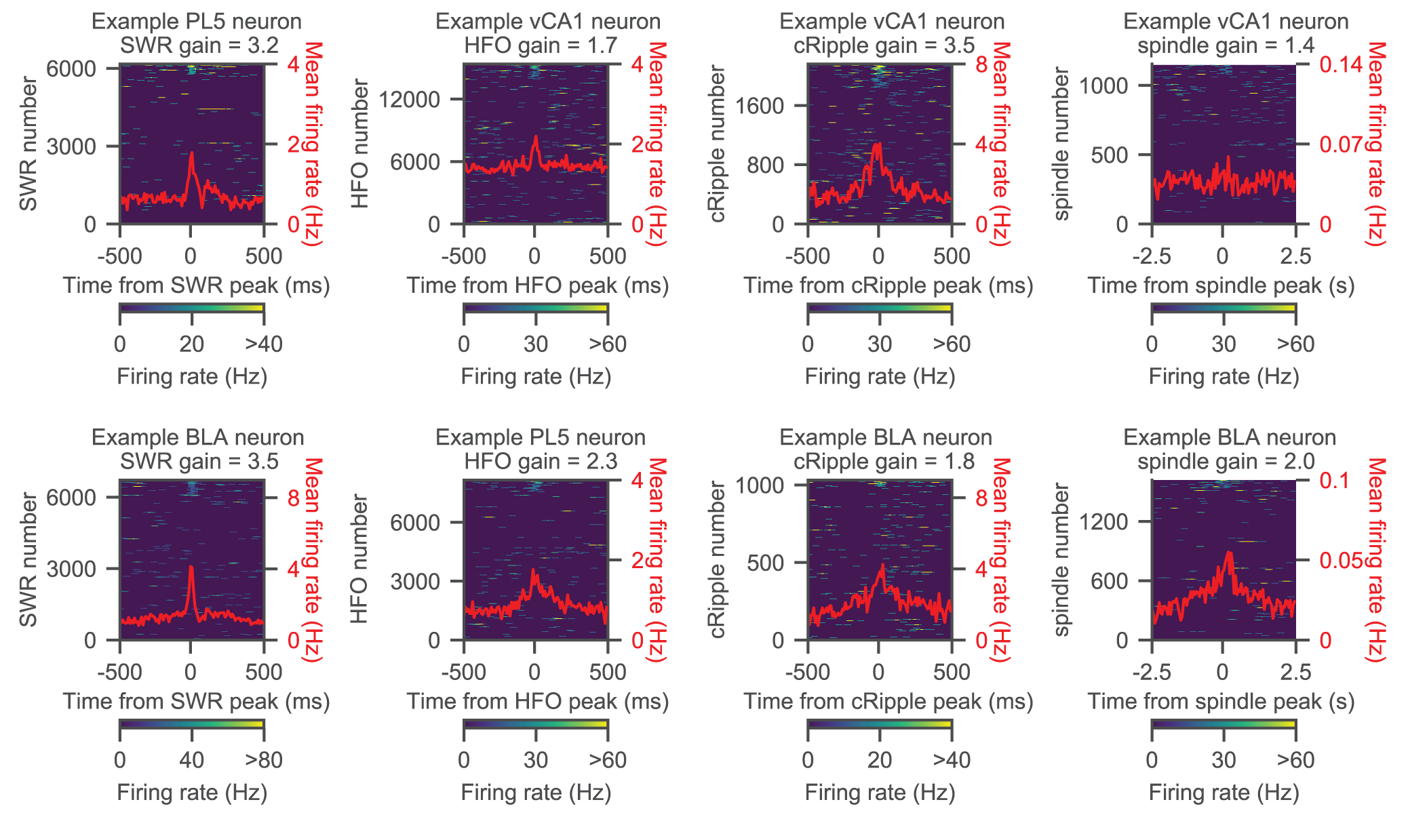

Supplement: Figure 3-1 — Additional examples of firing rates aligned to fast network oscillation events Firing rates (FRs) of example neurons aligned to power peaks of fast network oscillations (SWRs, HFOs, cRipples, and spindles). Similar to Fig. 3A, but these examples show the modulation of neuronal firing by oscillations detected in a different region than where the neuron was recorded. Peri-event FRs aligned with the peaks of individual oscillatory events are color-coded and sorted by FR peak. The red line represents the mean FR across oscillation events. The FR gain within the relevant oscillation is shown above each panel. Download Figure 3-1, TIF file. [file eneuro-12-ENEURO.0575-24.2025-s013.tif]

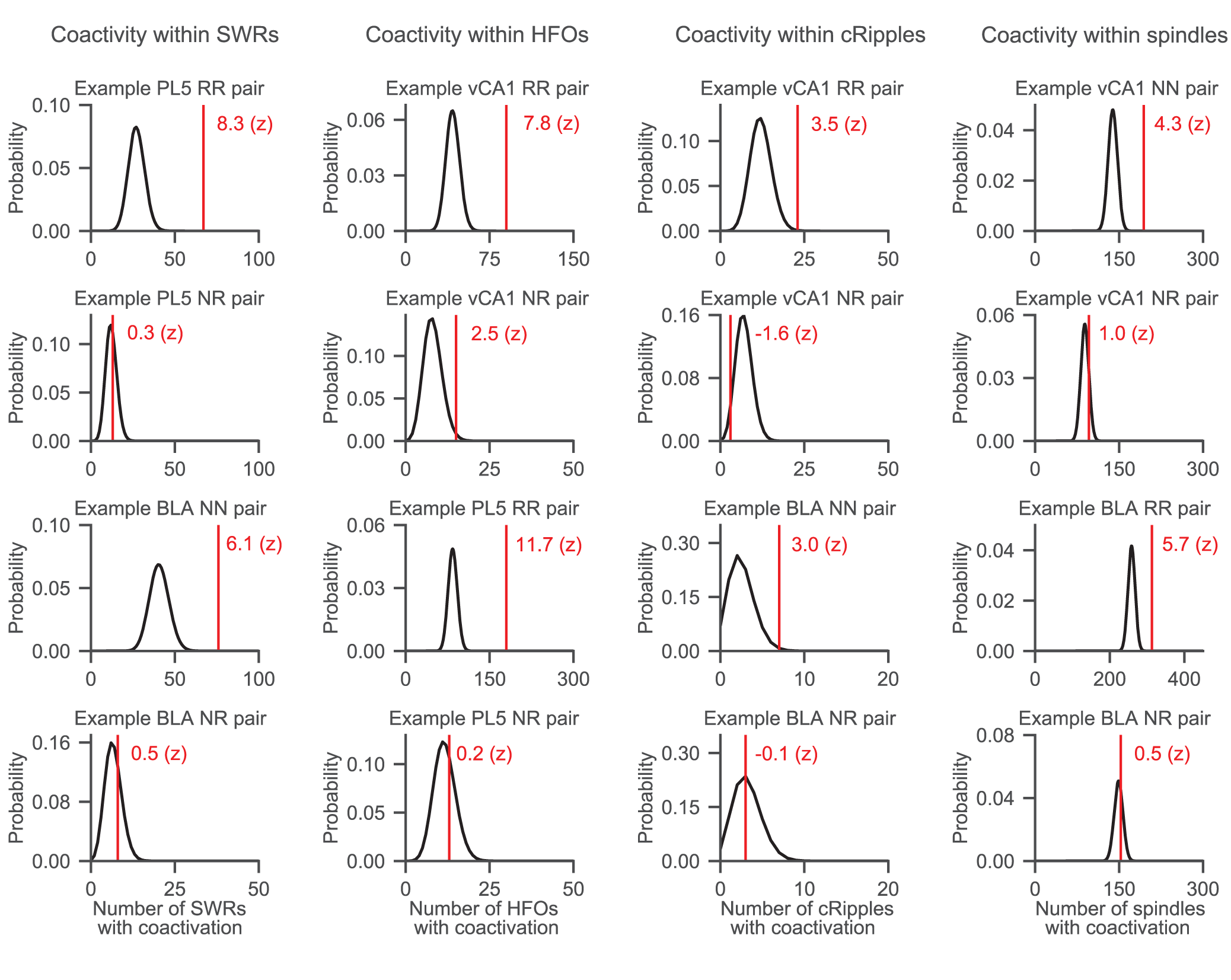

Supplement: Figure 4-1 — Additional examples of coactivation of neuron pairs during fast network oscillations Number of events in which example neuron pairs were coactivated, similar to Fig. 4A. Here, coactivation is shown during oscillations detected in a region different from where the neuron pair was recorded. The black curves show the theoretical distributions of the number of coactivation events, assuming that the neurons activate independently during oscillatory events. The observed number of coactivation events is indicated by the red vertical line, and the corresponding coactivity Z-score is displayed next to the line. Download Figure 4-1, TIF file. [file eneuro-12-ENEURO.0575-24.2025-s015.tif]

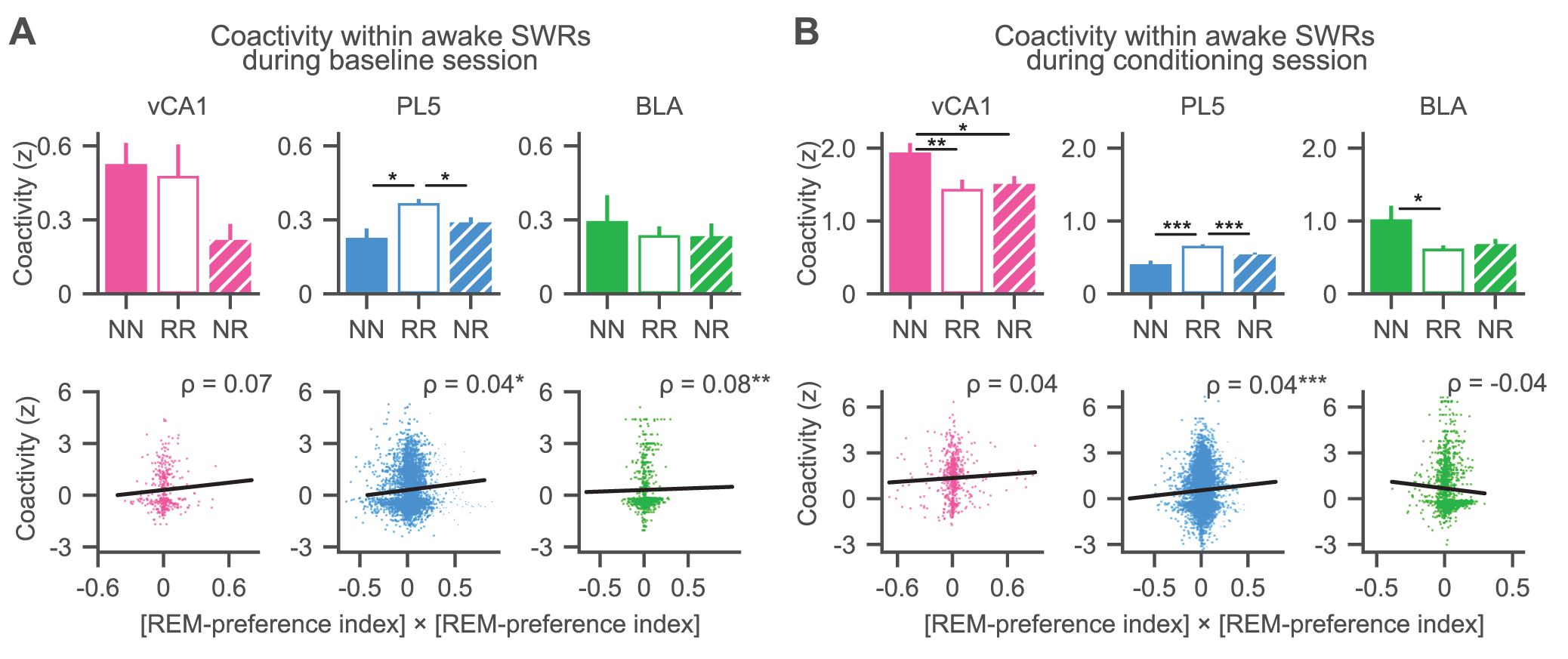

Supplement: Figure 7-1 — Within-region coactivity of excitatory neuron pairs during awake SWRs Within-region coactivity Z-scores for awake SWRs are shown separately for the baseline (A) and conditioning (B) sessions in vCA1, PL5, and BLA. Top panels: Mean coactivity Z-scores for each cell pair type. The NN, RR, and NR pairs are shown separately. Error bars represent standard errors of the mean (SEM). Bottom panels: Relationships between the products of REM-preference indices and coactivity Z-scores. Each dot represents a cell pair. Linear regression lines are shown in black, and the Spearman’s rank-order correlation coefficients are shown in the upper right. *p < 0.05, **p < 0.01, ***p < 0.001; Top panels: Post hoc Steel–Dwass test following the Kruskal–Wallis test. Bottom panels: Significance of Spearman’s rank-order correlation. Neuron pairs including non-significant neurons were also included in the correlation analysis. See Extended Data Fig. 7-2 for the number of cell pairs analyzed and detailed statistical results. Abbreviations: NN: NREM-preferring neuron pairs; RR: REM-preferring neuron pairs; NR: Pairs of NREM- and REM-preferring neurons. Download Figure 7-1, TIF file. [file eneuro-12-ENEURO.0575-24.2025-s019.tif]
