# Supplementary material for: Firing Activities of REM- and NREM-Preferring Neurons Are Differently Modulated by Fast Network Oscillations and Behavior in the Hippocampus, Prelimbic Cortex, and Amygdala
Source: eNeuro. 2025 May 23;12(5):ENEURO.0575-24.2025. doi: 10.1523/ENEURO.0575-24.2025 (PMC12118951; doi:10.1523/ENEURO.0575-24.2025)
Supplement: Figure 1-1 — Duration of NREM and REM sleep and wakefulness in the home cage (hc) and behavioral sessions Mean ± standard deviation of the duration and proportion of NREM sleep, REM sleep, and wakefulness during each home cage (hc) and behavioral session (n = 15 rats). Download Figure 1-1, DOCX file. [file eneuro-12-ENEURO.0575-24.2025-s002.docx]

**Extended Data Figure 1-1**

|  | NREM | REM | Awake |
| --- | --- | --- | --- |
| hc0 | 4,543 ± 1,443 s  (41.6 ± 13.4%) | 732 ± 544 s  (6.7 ± 5.1%) | 5,662 ± 1,924 s  (51.7 ± 17.3%) |
| hc1 | 4,888 ± 717 s  (50.4 ± 7.3%) | 999 ± 359 s  (10.3 ± 3.7%) | 3,801 ± 953 s  (39.2 ± 9.9%) |
| hc2 | 4,066 ± 1,311 s  (42.3 ± 13.6%) | 742 ± 440 s  (7.7 ± 4.6%) | 4,795 ± 1,604 s  (50.0 ± 16.8%) |
| hc3 | 5,505 ± 531 s  (57.6 ± 5.5%) | 1,365 ± 316 s  (14.3 ± 3.3%) | 2,674 ± 739 s  (28.0 ± 7.8%) |
| hc4 | 4,718 ± 932 s  (48.1 ± 9.4%) | 1,060 ± 465 s  (10.8 ± 4.7%) | 4,000 ± 1,237 s  (40.8 ± 12.7%) |
| Baseline | 26 ± 97 s  (2.4 ± 8.9%) | 0 s  (0%) | 1,065 ± 98 s  (97.7 ± 8.9%) |
| Conditioning | 0 s  (0%) | 0 s  (0%) | 2,748 s  (100%) |
| Context-retention | 0 s  (0%) | 0 s  (0%) | 240 s  (100%) |
| Cue-retention and extinction | 448 ± 613 s  (9.4 ± 12.8%) | 89 ± 184 s  (1.9 ± 3.8%) | 4,170 ± 818 s  (87.3 ± 17.1%) |
| Retention of extinction | 98 ± 205 s  (5.3 ± 11.0%) | 9.9 ± 37 s  (0.5 ± 2.0%) | 1,702 ± 256 s  (91.0 ± 13.8%) |
