# Supplementary material for: Firing Activities of REM- and NREM-Preferring Neurons Are Differently Modulated by Fast Network Oscillations and Behavior in the Hippocampus, Prelimbic Cortex, and Amygdala
Source: eNeuro. 2025 May 23;12(5):ENEURO.0575-24.2025. doi: 10.1523/ENEURO.0575-24.2025 (PMC12118951; doi:10.1523/ENEURO.0575-24.2025)
Supplement: Figure 1-2 — Number of NREM–REM–NREM triplets in extended sleep Total number of NREM–REM–NREM triplets and rats analyzed, related to Fig. 1B and D. Download Figure 1-2, DOCX file. [file eneuro-12-ENEURO.0575-24.2025-s003.docx]

**Extended data Figure 1-2**

|  | Analyses for excitatory neurons | | |  | Analyses for inhibitory neurons | | |
| --- | --- | --- | --- | --- | --- | --- | --- |
|  | vCA1 | PL5 | BLA |  | vCA1 | PL5 | BLA |
| Number of NREM–REM–NREM triplets | 147 triplets | 142 triplets | 197 triplets |  | 128 triplets | 142 triplets | 120 triplets |
| Number of examined rats | 8 rats | 8 rats | 12 rats |  | 7 rats | 8 rats | 7 rats |
