# Supplementary material for: Firing Activities of REM- and NREM-Preferring Neurons Are Differently Modulated by Fast Network Oscillations and Behavior in the Hippocampus, Prelimbic Cortex, and Amygdala
Source: eNeuro. 2025 May 23;12(5):ENEURO.0575-24.2025. doi: 10.1523/ENEURO.0575-24.2025 (PMC12118951; doi:10.1523/ENEURO.0575-24.2025)
Supplement: Figure 1-3 — Comparison of the number of NREM- and REM-preferring neurons and non-significant neurons defined by different classification thresholds To classify neurons as REM-preferring, NREM-preferring, or non-significant neurons, we generated a distribution of REM-preference indices from shuffled surrogates (REM/NREM bin label shuffling, n = 1,000) for each neuron (see Classification of REM- and NREM-preferring neurons in Materials and Methods). Throughout the paper, a neuron was labeled as REM- or NREM-preferring if its actual REM-preference index exceeded the 97.5th percentile or fell below the 2.5th percentile of the surrogate distribution, respectively (p < 0.05, 5% criterion). Otherwise, it was labeled as non-significant. Here, more stringent thresholds were tested for classifying REM- and NREM-preferring neurons (p < 0.01, 1% criterion; and p < 0.002, 0.2% criterion). Percentages in parentheses indicate the proportion of neurons initially classified using 5% criterion that also met the more stringent criterion. Download Figure 1-3, DOCX file. [file eneuro-12-ENEURO.0575-24.2025-s004.docx]

**Extended data Figure 1-3**

|  | NREM-preferring neuron | REM-preferring neuron | non-significant  neuron |
| --- | --- | --- | --- |
| vCA1 excitatory neurons |  | | |
| 5% criterion | 28 | 58 | 6 |
| 1% criterion | 26 (92.9%) | 58 (100%) | 8 |
| 0.2% criterion | 26 (92.9%) | 57 (98.3%) | 9 |
| PL5 excitatory neurons |  | | |
| 5% criterion | 85 | 303 | 30 |
| 1% criterion | 83 (97.6%) | 296 (97.7%) | 39 |
| 0.2% criterion | 83 (97.6%) | 293 (96.7%) | 42 |
| BLA excitatory neurons |  | | |
| 5% criterion | 41 | 159 | 9 |
| 1% criterion | 40 (97.6%) | 154 (96.9%) | 15 |
| 0.2% criterion | 37 (90.2%) | 154 (96.9%) | 18 |
| vCA1 inhibitory neurons |  | | |
| 5% criterion | 0 | 46 | 0 |
| 1% criterion | 0 (100%) | 46 (100%) | 0 |
| 0.2% criterion | 0 (100%) | 46 (100%) | 0 |
| PL5 inhibitory neurons |  | | |
| 5% criterion | 14 | 35 | 1 |
| 1% criterion | 14 (100%) | 34 (97.1%) | 2 |
| 0.2% criterion | 14 (100%) | 34 (97.1%) | 2 |
| BLA inhibitory neurons |  | | |
| 5% criterion | 3 | 15 | 0 |
| 1% criterion | 3 (100%) | 15 (100%) | 0 |
| 0.2% criterion | 3 (100%) | 15 (100%) | 0 |
