# Supplementary material for: Firing Activities of REM- and NREM-Preferring Neurons Are Differently Modulated by Fast Network Oscillations and Behavior in the Hippocampus, Prelimbic Cortex, and Amygdala
Source: eNeuro. 2025 May 23;12(5):ENEURO.0575-24.2025. doi: 10.1523/ENEURO.0575-24.2025 (PMC12118951; doi:10.1523/ENEURO.0575-24.2025)
Supplement: Figure 1-5 — Comparison of REM-preference indices between home cage sessions Spearman’s rank-order correlation coefficients of REM-preference indices between home cage sessions in the vCA1, PL5, and BLA, corresponding to Fig. 1H and Extended Data Fig 1-4. Download Figure 1-5, DOCX file. [file eneuro-12-ENEURO.0575-24.2025-s006.docx]

**Extended Data Figure 1-5**

| **Region** | **Number of cells** | **Comparison** | **ρ value** | **p value** |
| --- | --- | --- | --- | --- |
| vCA1 | 92 | hc0 vs hc1 | 0.517 | 1.328×10^−7^ |
|  |  | hc0 vs hc2 | 0.333 | 0.001 |
|  |  | hc0 vs hc3 | 0.418 | 3.384×10^−5^ |
|  |  | hc0 vs hc4 | 0.256 | 0.014 |
|  |  | hc1 vs hc2 | 0.391 | 1.165×10^−4^ |
|  |  | hc1 vs hc3 | 0.261 | 0.012 |
|  |  | hc1 vs hc4 | 0.291 | 0.005 |
|  |  | hc2 vs hc3 | 0.285 | 0.006 |
|  |  | hc2 vs hc4 | 0.270 | 0.009 |
|  |  | hc3 vs hc4 | 0.227 | 0.030 |
| PL5 | 418 | hc0 vs hc1 | 0.713 | 5.194×10^−66^ |
|  |  | hc0 vs hc2 | 0.402 | 1.172×10^−17^ |
|  |  | hc0 vs hc3 | 0.506 | 1.343×10^−28^ |
|  |  | hc0 vs hc4 | 0.423 | 1.572×10^−19^ |
|  |  | hc1 vs hc2 | 0.568 | 5.197×10^−37^ |
|  |  | hc1 vs hc3 | 0.640 | 1.585×10^−49^ |
|  |  | hc1 vs hc4 | 0.534 | 4.202×10^−32^ |
|  |  | hc2 vs hc3 | 0.629 | 2.006×10^−47^ |
|  |  | hc2 vs hc4 | 0.461 | 2.342×10^−23^ |
|  |  | hc3 vs hc4 | 0.772 | 8.874×10^−84^ |
| BLA | 209 | hc0 vs hc1 | 0.708 | 4.368×10^−33^ |
|  |  | hc0 vs hc2 | 0.473 | 4.859×10^−13^ |
|  |  | hc0 vs hc3 | 0.657 | 3.655×10^−27^ |
|  |  | hc0 vs hc4 | 0.681 | 7.481×10^−30^ |
|  |  | hc1 vs hc2 | 0.588 | 7.731×10^−21^ |
|  |  | hc1 vs hc3 | 0.695 | 1.886×10^−31^ |
|  |  | hc1 vs hc4 | 0.711 | 1.583×10^−33^ |
|  |  | hc2 vs hc3 | 0.574 | 9.965×10^−20^ |
|  |  | hc2 vs hc4 | 0.571 | 1.665×10^−19^ |
|  |  | hc3 vs hc4 | 0.796 | 6.188×10^−47^ |
