# Supplementary material for: Firing Activities of REM- and NREM-Preferring Neurons Are Differently Modulated by Fast Network Oscillations and Behavior in the Hippocampus, Prelimbic Cortex, and Amygdala
Source: eNeuro. 2025 May 23;12(5):ENEURO.0575-24.2025. doi: 10.1523/ENEURO.0575-24.2025 (PMC12118951; doi:10.1523/ENEURO.0575-24.2025)
Supplement: Figure 1-7 — Spearman’s rank-order correlation coefficients between REM-preference indices of different home cage sessions evaluated using bootstrapping Statistical details for Extended Data Fig. 1-6. As in Extended Data Fig. 1-5, this table presents Spearman’s rank-order correlation coefficients (ρ) of REM-preference indices between different home cage sessions, estimated using bootstrapping. The median correlation coefficient and the p-value against the null hypothesis (that the correlation coefficient is zero) are reported. Download Figure 1-7, DOCX file. [file eneuro-12-ENEURO.0575-24.2025-s008.docx]

**Extended Data Figure 1-7**

| **Region** | **Comparison** | **ρ value (median)** | **p value** |
| --- | --- | --- | --- |
| vCA1 | hc0 vs hc1 | 0.514 | < 0.001 |
|  | hc0 vs hc2 | 0.331 | < 0.001 |
|  | hc0 vs hc3 | 0.415 | < 0.001 |
|  | hc0 vs hc4 | 0.254 | 0.007 |
|  | hc1 vs hc2 | 0.387 | < 0.001 |
|  | hc1 vs hc3 | 0.262 | 0.011 |
|  | hc1 vs hc4 | 0.290 | 0.004 |
|  | hc2 vs hc3 | 0.283 | 0.007 |
|  | hc2 vs hc4 | 0.267 | 0.010 |
|  | hc3 vs hc4 | 0.226 | 0.020 |
| PL5 | hc0 vs hc1 | 0.713 | < 0.001 |
|  | hc0 vs hc2 | 0.400 | < 0.001 |
|  | hc0 vs hc3 | 0.503 | < 0.001 |
|  | hc0 vs hc4 | 0.422 | < 0.001 |
|  | hc1 vs hc2 | 0.567 | < 0.001 |
|  | hc1 vs hc3 | 0.640 | < 0.001 |
|  | hc1 vs hc4 | 0.535 | < 0.001 |
|  | hc2 vs hc3 | 0.628 | < 0.001 |
|  | hc2 vs hc4 | 0.461 | < 0.001 |
|  | hc3 vs hc4 | 0.770 | < 0.001 |
| BLA | hc0 vs hc1 | 0.707 | < 0.001 |
|  | hc0 vs hc2 | 0.470 | < 0.001 |
|  | hc0 vs hc3 | 0.656 | < 0.001 |
|  | hc0 vs hc4 | 0.682 | < 0.001 |
|  | hc1 vs hc2 | 0.586 | < 0.001 |
|  | hc1 vs hc3 | 0.694 | < 0.001 |
|  | hc1 vs hc4 | 0.711 | < 0.001 |
|  | hc2 vs hc3 | 0.574 | < 0.001 |
|  | hc2 vs hc4 | 0.570 | < 0.001 |
|  | hc3 vs hc4 | 0.794 | < 0.001 |
