# Supplementary material for: Firing Activities of REM- and NREM-Preferring Neurons Are Differently Modulated by Fast Network Oscillations and Behavior in the Hippocampus, Prelimbic Cortex, and Amygdala
Source: eNeuro. 2025 May 23;12(5):ENEURO.0575-24.2025. doi: 10.1523/ENEURO.0575-24.2025 (PMC12118951; doi:10.1523/ENEURO.0575-24.2025)
Supplement: Figure 1-9 — Comparison of Spearman’s rank-order correlation coefficients of REM-preference indices Statistical details for Extended Data Fig. 1-8. This table compares Spearman’s rank-order correlation coefficients (ρ) for REM-preference indices between temporally adjacent home cage (hc) sessions. Top: Statistical details for Extended Data Fig. 1-8A. Comparison of correlation coefficients between consecutive hc session pairs within each brain region. Bottom: Statistical details for Extended Data Fig. 1-8B. Comparison of correlation coefficients between brain regions. Bootstrapping was used to estimate the 95% confidence intervals (CIs) of the differences in correlation coefficients for both comparisons. The Spearman’s correlation coefficient of REM-preference indices between sessions hcX and hcY is denoted as ρXY. Download Figure 1-9, DOCX file. [file eneuro-12-ENEURO.0575-24.2025-s010.docx]

**Extended data Figure 1-9**

| Region | Compared ρ | 95% CI | P value |
| --- | --- | --- | --- |
| vCA1 |  |  |  |
|  | ρ_01_ – ρ_12_ | [-0.117, 0.367] | 0.851 |
|  | ρ_12_ – ρ_23_ | [-0.187, 0.387] | 0.759 |
|  | ρ_23_ – ρ_34_ | [-0.251, 0.358] | 0.639 |
| PL5 |  |  |  |
|  | ρ_01_ – ρ_12_ | [-0.068, 0.359] | 0.907 |
|  | ρ_12_ – ρ_23_ | [-0.290, 0.174] | 0.304 |
|  | ρ_23_ – ρ_34_ | [-0.337, 0.045] | 0.065 |
| BLA |  |  |  |
|  | ρ_01_ – ρ_12_ | [-0.089, 0.329] | 0.864 |
|  | ρ_12_ – ρ_23_ | [-0.208, 0.235] | 0.540 |
|  | ρ_23_ – ρ_34_ | [-0.414, -0.035] | 0.012 |
| Region pair | Compared ρ | 95% CI | P value |
| vCA1 – PL5 |  |  |  |
|  | ρ_01_ | [-0.391, 1.645×10^−5^] | 0.025 |
|  | ρ_12_ | [-0.439, 0.082] | 0.090 |
|  | ρ_23_ | [-0.609, -0.070] | 0.006 |
|  | ρ_34_ | [-0.780, -0.302] | <0.001 |
| vCA1 – BLA |  |  |  |
|  | ρ_01_ | [-0.386, 0.019] | 0.035 |
|  | ρ_12_ | [-0.444, 0.049] | 0.060 |
|  | ρ_23_ | [-0.558, -0.018] | 0.017 |
|  | ρ_34_ | [-0.795, -0.332] | <0.001 |
| PL5 – BLA |  |  |  |
|  | ρ_01_ | [-0.180, 0.195] | 0.523 |
|  | ρ_12_ | [-0.250, 0.211] | 0.441 |
|  | ρ_23_ | [-0.169, 0.278] | 0.678 |
|  | ρ_34_ | [-0.174, 0.122] | 0.385 |
