# Supplementary material for: Firing Activities of REM- and NREM-Preferring Neurons Are Differently Modulated by Fast Network Oscillations and Behavior in the Hippocampus, Prelimbic Cortex, and Amygdala
Source: eNeuro. 2025 May 23;12(5):ENEURO.0575-24.2025. doi: 10.1523/ENEURO.0575-24.2025 (PMC12118951; doi:10.1523/ENEURO.0575-24.2025)
Supplement: Figure 2-2 — Duration and occurrence rate of network oscillatory events The duration (median with 1st and 3rd quartiles) and occurrence rate (mean ± standard deviation) of fast network oscillations detected in vCA1 (sleep and awake SWRs), PL5 (cRipples and spindles), and BLA (HFOs) recordings, and the number of rats in which the respective oscillatory events were examined. Download Figure 2-2, DOCX file. [file eneuro-12-ENEURO.0575-24.2025-s012.docx]

**Extended Data Figure 2-2**

|  | Duration (ms) | | |  | Occurrence rate (1/s) | Number of examined rats |
| --- | --- | --- | --- | --- | --- | --- |
|  | First quartile | Median | Third quartile | Number of events |  |  |
| Sleep SWR | 40.0 | 52.0 | 68.0 | 64023 | 0.328 ± 0.063 | 8 |
| HFO | 36.0 | 44.8 | 60.0 | 116643 | 0.438 ± 0.111 | 11 |
| cRipple | 54.4 | 60.8 | 74.4 | 13489 | 0.070 ± 0.021 | 8 |
| spindle | 412.8 | 502.4 | 653.6 | 10942 | 0.056 ± 0.008 | 8 |
| Awake SWR | 42.4 | 56.8 | 79.2 | 14754 | 0.060 ± 0.014 | 8 |
